# Undergraduate nursing students’ knowledge and attitudes towards Primary Health Care

**DOI:** 10.1371/journal.pgph.0007178

**Published:** 2026-09-01

**Authors:** Nafa Odeh, Rabia S. Allari

**Affiliations:** 1 Faculty of Nursing/Al-Ahliyya Amman University, Amman, Jordan; 2 Hamad Medical Corporation/Qatar, Doha, Qatar; National Center for Chronic and Noncommunicable Disease Control and Prevention, Chinese Center for Disease Control and Prevention, CHINA

## Abstract

This study aimed to assess undergraduate nursing students’ knowledge and attitudes toward Primary Health Care in Jordan. This study adopted a descriptive cross-sectional design, involving 157 nursing students from two private and two public universities in the central region of Jordan. It utilized an Arabic version of a self-administered online Primary Health Care Questionnaire. The findings indicated that the majority of students exhibited high knowledge (77%) and positive attitudes (97%); however, there was a significant difference between students’ knowledge, attitudes, and their socio-demographic factors. Moreover, the results revealed that demographic factors affect primary healthcare attitudes and knowledge, indicating that students’ perspectives concerning PHC can be influenced by demographic factors, including gender (B = 0.173), academic performance (B = 0.044), and intent to leave nursing in the future (B = 0.122) at a significance level (<0.05). Furthermore, the findings demonstrated that these demographic and academic factors significantly predicted the attitudes of undergraduate nursing students toward PHC in Jordan. Nursing students demonstrated high knowledge and positive attitudes toward primary healthcare services. Selected demographic characteristics were significantly associated with knowledge and attitudes, as supported by the statistical analysis. These findings highlight the importance of strengthening primary healthcare content and practical learning experiences within nursing education to support students’ preparedness for future community-based health promotion and disease-prevention roles.

## Introduction

Primary HealthCare (PHC) is defined by the World Health Organization [1] as the delivery of specific health services using technologies and procedures that are both socially and scientifically acceptable. PHC’s principal goal is to ensure that every individual and family living in a particular community has access to comprehensive healthcare, with an emphasis on prevention, health promotion, treatment, and rehabilitation. According to WHO [1], PHC is responsible for ensuring accessible and equitable health services for all, promoting preventive health programs, managing chronic conditions, coordinating care across healthcare levels, and empowering communities in health education and decision-making to achieve enhanced health outcomes As the mainstay of public health services and the most observable and essential part of the country’s healthcare system, PHC receives the most attention.

Furthermore, PHC is essential for healthcare sustainability and the community’s social and economic progress. The most common form of interaction between individuals, families, and communities with the organized health system concerns the delivery of health services at residential homes and workplaces [1]. As Nnamuchi and Ilodigwe [2] note, by emphasizing the global focus on social justice and highlighting the crucial role of PHC in providing adequate healthcare internationally, the Alma-Ata declaration (reported in 1978) marked a significant milestone in public healthcare.

Over time, health systems throughout the globe have developed; therefore, the PHC systems have also evolved. Its official adoption and continuous development can be observed in later international health initiatives, such as the Astana Declaration of 2018 [3]. This specific proclamation reaffirmed the international emphasis on PHC as the most advantageous and successful means to meet universal health services coverage and address the associated Sustainable Development Goals (SDGs) [3]. As stated by WHO [1], appropriate applications of PHC can address approximately 90% of an individual’s lifetime health needs; however, despite the considerable progress that has been made towards achieving the goal of global coverage and efficiency, some barriers persist.

PHC nurses play a key role in providing both direct care for patients and essential services that cater to the health requirements of a broad range of patient populations [4,5]. Primarily, nurses are responsible for up to 80% of PHC patients, which highlights the critical role they play in the healthcare system [6,7]. Nurses serve a critical function in advancing community health by delivering primary healthcare services. Their professional responsibilities encompass health education, disease prevention, health promotion, early detection of health issues, and facilitating access to appropriate primary healthcare for individuals and communities. Consequently, nursing students’ knowledge, attitudes, and practices regarding primary healthcare are significant, as these factors may affect their readiness to participate effectively in community-based health promotion and prevention initiatives [8].

Nurses are essential to cultivating health literacy: they teach patients to take preventive actions, manage chronic diseases, and make lifestyle changes. Nurses working in PHC settings are reported to contribute approximately 33% of their time to educational interventions aimed at improving patient outcomes [9–11]. Additionally, nurses are essential in coordinating patient care across various health systems, helping to implement coordinated care plans that have resulted in a 20% reduction in hospital readmissions [12,13].

In this context, nursing education is essential for preparing future healthcare professionals, particularly in the PHC industry. To equip students for their responsibilities in PHC, where they manage most patient encounters, nursing schools must provide proper training [9]. Contemporary research has identified a concerning trend in nursing education programs whereby greater emphasis is placed on acute-care settings rather than PHC. This imbalance could leave students underprepared for PHC roles. Despite the recognized importance of PHC within the larger healthcare system, this gap highlights the need for educational reforms that better incorporate core healthcare skills into nursing curricula [9].

Upon transferring from classroom settings to actual healthcare facilities, nursing students encounter several notable challenges. When embarking on their careers in this sector, nursing students frequently exhibit varying levels of preparedness, which might affect their confidence in PHC environments. According to research conducted by Aslan and Pekince [14], nursing students typically exhibit a positive attitude towards primary health; however, their actual ability to manage PHC activities may differ. A previous study among Australian nursing students showed that only 22.8% of students stated that they would prefer to work in PHC once they graduate, which suggests a misalignment between their educational background and their professional aspirations [15]. Furthermore, there is a discernible variation in the students’ preparedness for undertaking PHC responsibilities. For example, Khattak et al. [10] reported that 40% of students believe they are well equipped to conduct their regular PHC responsibilities once their schooling is complete.

Additionally, students’ scholarly interest in PHC throughout nursing school has a significant impact on their intentions and attitudes toward continuing a career in this sector. Current evidence indicates that students who are exposed to numerous PHC environments throughout their training are more likely to select PHC as their post-graduation career path [16].To guarantee that nursing graduates are not only motivated but also adequately equipped to assume PHC responsibilities, substantial improvements should be made to the way PHC-specific knowledge and skills are currently incorporated into nursing education.

Educational programs for nursing students play a key role in providing them with the competencies and knowledge required to perform their duties at PHC. PHC courses in nursing education are designed to provide students with the fundamental knowledge and competencies they require (such as active community involvement, cultural sensitivity, and effective patient communication) [17]. Despite the critical role PHC plays in healthcare, contemporary research has revealed that nursing curricula frequently have a narrow focus, which results in students being unprepared for PHC responsibilities [18,19]. Following the completion of their training, approximately 40% of nursing students believe that they are adequately prepared to oversee PHC activities; therefore, the career paths of students may be greatly impacted by incorporating PHC experience into the curriculum [10].

Moreover, providing comprehensive training for PHC clinical courses can greatly increase students’ desire to work within a PHC setting [15,16]; therefore, students who receive comprehensive PHC training are more likely to believe that they are proficient in critical PHC skills (such as cultural sensitivity and effective patient communication). Additionally, it has been reported that incorporating practical PHC experiences into nursing education enhances students’ preparedness for performing subsequent PHC work and duties [19], which reinforces how existing teaching methods differ from the evolving demands of healthcare delivery. Furthermore, this discrepancy emphasizes the need for urgent educational reforms that prioritize PHC competency development. Such reforms will also ensure that nursing students are sufficiently equipped to negotiate the complex healthcare environment [9].

Numerous factors have a significant impact on nursing students’ preparedness and interest in conducting PHC duties after graduation; therefore, it is imperative to develop an understanding of their knowledge and attitudes regarding PHC. Consistently, previous research has reported that nursing students around the world have different attitudes and knowledge levels. For example, Khattak et al. [10] found that medical students in Pakistan possessed a moderate level of knowledge, which suggests that they lack a thorough understanding of fundamental healthcare. According to Calma et al. [9], a curriculum that focuses primarily on acute care may have an impact on students’ readiness to interact with PHC. Given that nursing schools do not sufficiently incorporate or contain PHC components, this trend points to a substantial educational gap. Consequently, students’ opinions concerning careers in PHC are greatly influenced.

Nursing students demonstrate deviations in both their educational patterns and their perceptions of PHC. For example, globally, Bloomfield et al.’s [15] study findings emphasize the complexity of enhancing PHC education, necessitating curriculum adjustments to align more effectively with the specific requirements and circumstances of PHC in diverse healthcare environments. Nationally, a study conducted in Jordan by Abu-Baker et al. [6] observed mostly positive views towards health promotion, a crucial component of basic healthcare.

Primary health care in Jordan is described as necessary, easily available, community-based care provided at the initial point of contact via a network of facilities (such as village clinics, primary health centers, and comprehensive health centers) governed by the Ministry of Health. Delivered by multidisciplinary teams comprising doctors, nurses, and midwives, Jordanian PHC prioritizes health promotion, disease prevention, maternity and child health, immunization, and basic curative treatments [20]. Nationally, the PHC is defined as essential health care that is universally accessible, scientifically sound, and delivered by a trained workforce via community participation and integrated referral systems [20].

The arguments and practices in Jordan indicate a growing interest in PHC among university students, which is driven by both institutional and academic efforts to enhance attitudes towards PHC. Research has demonstrated that public health programs and university courses play a pivotal role in heightening students’ health awareness [21]. The students relied heavily on the information provided by these programs and courses (primarily via official websites and social media platforms), which contributed to modifying their health behaviors and practices. When developing academic initiatives to promote PHC, Jordanian universities (in collaboration with local and international universities and with support from foreign parties) launched a professional diploma in PHC. This program aims to train general practitioners and family physicians, reflecting a growing academic interest in this field. While this initiative is physician-focused, it highlights broader institutional efforts that may indirectly influence the development of PHC-related education across health disciplines, including nursing. Additionally, the universities organized several scientific conferences concerning PHC-related issues (such as health and well-being), which discussed the shift towards PHC and comprehensive health insurance, thereby demonstrating the role of universities in promoting this trend [22].

Furthermore, efforts by academic institutions to promote health awareness succeed via the involvement of academic bodies (such as faculty members) in nursing departments’ initiatives to encourage students to receive primary and preventive healthcare services, thereby activating their role as active agents in improving health conditions [23]. However, such undertakings require the effective planning of health awareness activities, implementing them with learners, and enlisting additional volunteers. When supported by academic and community initiatives aimed at promoting health awareness and developing skills in this area, there is a noticeable trend among university students toward PHC [24].

The PHC system significantly impacts the overall health outcomes in Jordan; therefore, gaining insight concerning the knowledge and attitudes of nursing students towards PHC is of the utmost importance. Although previous studies established the foundation for comprehending health promotion and awareness in Jordan, the current study is elevated by its specific focus on the professional readiness of the future nursing workforce. Although previous Jordanian studies have examined university students’ general health awareness [21] and favorable opinions of health promotion [6], this study is the first to specifically evaluate undergraduate nursing students’ attitudes and knowledge regarding the Primary Healthcare system. By focusing on this specific professional group, this research addresses the significant gap between institutional physician-led activities and the nursing workforce’s actual readiness to accomplish national healthcare goals.

This study may help in the preparedness of nursing students in Jordan to actively engage in the PHC system, an area that has been mostly overlooked by contemporary empirical research. This study seeks to assess these elements to not only highlight the current state of PHC education but also provide ideas for enhancing the curriculum to effectively equip future nurses to meet the healthcare needs of the Jordanian community. Furthermore, this research seeks to contribute to the broader goals of improving health service delivery and developing a robust healthcare system in Jordan, which is consistent with both domestic health legislation and global health standards. Therefore, the aim was to assess undergraduate nursing students’ knowledge and attitudes toward PHC in Jordan. According to this researcher’s knowledge, no study in Jordan has assessed undergraduate nursing students’ knowledge and attitudes towards PHC; therefore, this research is the first of its kind in Jordan and the first to address the current research gap.

### Study objectives

The study aimed to assess the undergraduate nursing students’ knowledge and attitudes towards PHC in Jordan. In alignment with this aim, the study pursues the following research objectives:

1. To assess the level of knowledge regarding PHC among undergraduate nursing students in Jordan.
2. To assess the levels of attitudes towards PHC among undergraduate nursing students in Jordan.
3. To assess the association between knowledge scores, level of attitudes, socio-demographics, and academic-related factors among undergraduate nursing students in Jordan.
4. To identify the significant factors that predict the level of attitudes towards PHC among undergraduate nursing students in Jordan.

## Materials and methodologies

### Ethical statement

The PHCQ study complies with all relevant ethical standards, ensuring participant safety and dignity. All of the necessary approvals were provided by the relevant institutions. Additionally, the researcher emphasized the participants’ right to confidentiality, the voluntary nature of participation, and the option to withdraw at any point without penalty. Furthermore, by choosing to continue with the electronic questionnaire, each of the participants provided their implied informed consent. No special permission was required to use the selected study tool because it was publicly available online. To ensure compliance with ethical guidelines, the study obtained approval from Al-Ahliyya Amman University’s Institutional Review Board (IRB) before starting (MM 2/6–2024). Data was anonymized and securely stored to protect participant privacy. All procedures adhere to ethical norms for research involving human subjects, ensuring transparency, responsibility, and respect for participants’ rights and well-being. Additionally, strict protocols were followed for data storage. After the study, data were securely stored on the researcher’s laptop, with access limited to authorized study team members. This process safeguarded participant information, thereby maintaining the accuracy and confidentiality of the data collected.

### Study design

In this study, data concerning the knowledge and attitudes of Jordanian nursing students regarding PHC were collected via the use of a descriptive cross-sectional research method. This approach facilitates an examination of knowledge and attitudes at a specific point in time and allows for data collection from different participant groups [25]; therefore, it is considered suitable for analyzing the research questions and understanding the current state of PHC knowledge and attitudes among Jordanian nursing students.

### Setting

The study was conducted at four universities (two private and two governmental) located in the central region of Jordan. The selection of governmental and private universities provided a robust setting for assessing a diversity of student experiences. These universities are known for their sizable enrolments and comprehensive nursing curricula that incorporate PHC as a crucial component of community nursing courses. A multi-stage sampling approach was employed: the four universities were selected via stratified random sampling (governmental vs. private), followed by convenience sampling of eligible nursing students within each selected institution. These universities were chosen using clustered stratified random sampling, whereby the four universities were selected randomly to guarantee a thorough assessment of PHCs’ knowledge and attitudes across various nursing educational programs and reinforce the study’s investigation of the variables affecting nursing students’ knowledge and attitudes towards PHC [26].

### Population and sample

This study’s target population consisted of undergraduate nursing students in Jordan. The accessible population included nursing students enrolled at selected private and government universities across Jordan, from which the sample was drawn to provide a diverse and representative cross-section of future healthcare professionals in the country. This study employed a convenience sampling method to select participants who met specific criteria: firstly, they must be currently enrolled as undergraduate nursing students at one of the chosen universities in central Jordan, and secondly, they must be in their third year of study (or higher). Typically, courses covering primary health care concepts begin in the third year; therefore, the second criterion ensures that the participants have been exposed to a significant portion of the nursing curriculum, including primary health care components. Participation was voluntary and based on informed consent. The questionnaire was administered in Arabic; therefore, to ensure a clear understanding, only students who were proficient in Arabic were selected. The recruitment process took place between 04/06/2024 and 30/07/2024.

Using G*Power analysis [27] for correlation and regression with an effect size of 0.15, power of 0.8, alpha of 0.05, and 13 predictors, the calculated sample size is 131. To account for potential dropouts and ensure robustness, an additional 20% was added, resulting in a final sample size of 157 participants. This approach ensures sufficient statistical power to detect significant effects and accurately represent the population under study.

### Study instrument

The study utilized a self-administered questionnaire consisting of two sections:

**Section One:** Sociodemographic variables including age, gender, university type, monthly family income, and “intent to leave nursing in the future,” (defined as students’ self-reported plan to leave the nursing profession entirely after graduation, rather than seeking further training or specialization within health care). Additionally, this section included academic-related variables encompassing academic performance, represented by the Grade Point Average (GPA); the number of completed credits at the time of the study; and hours spent studying per week. These variables were selected to provide a comprehensive understanding of the elements affecting nursing students’ knowledge and attitudes towards PHC [9,13].

**Section Two:** The Primary Health Care Questionnaire (PHCQ) (developed by [28] is intended to assess the knowledge and attitudes of nursing students toward PHC. The PHCQ’s dimensions (such as community orientation, health education, prevention, and accessibility) are essential to PHC practice in Jordan and are represented in both training and service delivery. For example, PHC services in Jordan consist of immunization and the health of mothers and children, disease prevention and health education, basic referral and curative care systems, and community-focused services. Nursing education and clinical training in PHC settings have always placed a robust emphasis on these topics.

*Knowledge items,* which consisted of 35 items (such as the significance of accessibility, the function of technology, and the range of services offered within PHC frameworks), are formatted as True/False questions. All answers should be true according to the scoring system of the questionnaire. The scores are converted to percentages (ranging from 0% to 100%) to enhance the clarity of the results [28]. Scores below one standard deviation from the mean, or less than 66.36 for knowledge, are considered low or less favorable; therefore, they are used as cut points for interpretation. Following this, the knowledge scale result was categorized according to one of the following three levels: high knowledge (≥ 66.36%), moderate knowledge (50% < 66.36%), and low knowledge (< 50%). *The attitude scale* consists of 34 items presented in a 4-point Likert scale (strongly disagree, disagree, agree, and strongly agree). For items with negative meaning, the study reversed item codes using SPSS and placed a symbol (R) beside them. Subsequently, the scores were converted to percentages (ranging from 0% to 100%) to enhance their clarity. Scores below one standard deviation from the mean, or less than 65.91 for attitudes, are considered low or less favorable; therefore, they are used as cut points for interpretation. The scoring system of this scale is categorized into three levels: highly positive attitudes (≥ 65.91%), moderate attitudes (65.91% - 50%), and less positive attitudes (< 50%). Due to its robust psychometric qualities, PHCQ facilitates an effective evaluation of participant attitudes and knowledge concerning PHC. Test-retest reliability measures were robust and demonstrated good stability over time, with Spearman’s correlations of 0.67 for knowledge and 0.76 for attitudes [28].

An exacting forward-backwards translation procedure was used to guarantee the accuracy and consistency of the Arabic translation of the PHCQ. This approach, which aligns with Beaton et al. [29], is renowned for generating translations that are both linguistically and culturally appropriate and adhere to established procedures.

Following the translation process, seven expert panel reviews were conducted to finalize the Arabic version of the PHCQ (see S1 Text) and to validate the instrument (face validity). The panel consisted of four translators, the principal researcher, and two additional experts in nursing education and public health with extensive experience in questionnaire design and cultural adaptation. This review focused on resolving any remaining ambiguities, ensuring the questionnaire’s cultural appropriateness, and verifying that the language was clear and understandable for the Jordanian target audience. A pilot study involving 15 nursing students (10% of the initial study population) was conducted to assess the Arabic version of PHC, specifically the time required to complete the questionnaire, and its usability, clarity, and reliability. The results indicated that Cronbach’s alpha was 0.85 for the knowledge items and 0.88 for the attitude measures, demonstrating good dependability and strong internal consistency.

### Data collection procedure

The researcher prepared the instrument using Google Forms, an easy-to-navigate online tool that simplifies data collection and management. Between 04/06/2024 and 30/07/2024, the researcher gathered data from the targeted participants. The process of editing the instrument in Google Forms involved creating a comprehensive online survey based on PHCQ. The researcher approached the nursing faculty at the targeted universities to discuss the questionnaire and obtain their approval for distribution. After presenting the questionnaire to the deans, the researcher requested their assistance in encouraging nursing students to share the questionnaire link via faculty members and university resources (such as student portals or official social media groups). To increase engagement and provide a more interactive explanation of the study’s goal, the researcher also sought permission to interact directly with students on campus. After receiving approval, the researcher distributed the Google Forms link via official email channels, social media (WhatsApp), or other university-approved communication tools. The questionnaire included a brief introduction concerning the study’s purpose, assurances of confidentiality and anonymity, and instructions highlighting the voluntary nature of participation. Participants were informed that the questionnaire would take twenty minutes to complete and reassured that their responses would be stored securely and used solely for research purposes.

To enhance response rates, periodic reminders were sent to each participant, and the accessibility of Google Forms enabled participants to complete the questionnaire at their convenience on any device with internet access. Following the completion of the data collection phase, the researcher exported the data from Google Forms to a password-protected spreadsheet for analysis.

In addition to expediting the data collection process, this configuration reduced data entry errors and promoted effective data management and analysis, all of which support the study’s objectives concerning a precise evaluation of nursing students’ attitudes and knowledge regarding PHC.

### Statistical analysis

The data collected from the questionnaire was carefully managed to ensure accuracy and confidentiality. Data entry was conducted using SPSS version 26. The dataset underwent a thorough cleaning process to address any inconsistencies, missing values, or outliers. Descriptive checks were performed to verify that all data was filled out within the expected range of values. To prepare the data for analysis, the dataset was tested for normality using statistical measures (such as the Shapiro-Wilk test and a visual inspection of histograms and Q-Q plots).

The initial run of data analysis related to the normality test using Shapiro-Wilk through SPSS showed that the data was not normally distributed with a significance level of (p < 0.05). To address this issue, the study applied an approach of transforming the means of the variables to ensure normal distribution of the data and go beyond further parametric analyses. Moreover, the new results achieved the normality assumption using a similar test with a significance level of (p < 0.05). In the first instance, the sample’s socio-demographic and academic-related characteristics were summarized using descriptive statistics (mean, standard deviation, and frequency), thereby providing a clear picture of the participant pool. Furthermore, the associations between sociodemographic and academic-related variables and knowledge and attitudes were also examined via Pearson’s chi-square correlation. To assess variations in knowledge and attitudes, mean scores based on categorical variables, an independent t-test, and one-way ANOVA were utilized. Following this, hierarchical regression analysis was applied to identify predictors of attitudes. This approach facilitated the controlled assessment of how several factors (such as age and academic performance) influence the primary outcomes, adjusting for potential confounders sequentially. All tests were conducted with a significance threshold set at p < 0.05, ensuring that findings are statistically clear and provide meaningful insights into the factors that predict nursing students’ attitudes toward PHC.

## Results

### Socio-demographic participants

In this study, 157 participants completed and returned the questionnaires (Table 1). More than half of the participants were male (n = 90, 57.3%), and 67 participants were female (42.7%). The age of the participants was varied; therefore, the findings revealed that two-thirds (n = 117, 74.5%) of the participants were between 20 and 25 years old. Furthermore, the majority of participants were enrolled in private universities (n = 102, 65%). Approximately two-thirds of the participants (n = 123, 78.3%) reported having no intent to leave nursing in the future, while the remaining participants (n = 34, 21.7%) reported they have an intent to leave nursing in the future.

**Table 1:** Socio-demographics and academic-related data of the sample (N = 157).

Variable	Options	N	%
Age	20-25 years	117	74.5
	25 < -30 years	33	21.0
	Above 30 years	7	4.5
Monthly family income	250-500 JOD	61	38.8
	501-750 JOD	60	38.2
	751-1000 JOD	29	18.5
	Above 1000 JOD	7	4.5
Academic Performance (GPA)	Fair	24	15.3
	Good	73	46.5
	Very good	43	27.4
	Excellent	17	10.8
Number of Completed Credits	60-80 hours	13	8.3
	81-100 hours	60	38.2
	101-120 hours	77	49.0
	Above 120 hours	7	4.5
Hours Spent Studying per Week	<10 hours	25	15.9
	10-20 hours	102	65.0
	21-30 hours	30	19.1
Gender	Male	90	57.3
	Female	67	42.7
University type	Governmental	55	35.0
	Private	102	65.0
Intent to leave nursing in the future	Yes	34	21.7
	No	123	78.3

N: Number, %: Percentage.

### The PHCQ (Knowledge scale)

The items that measure PHCQ (Knowledge scale) are presented in Table 2. The scoring system of this scale is categorized into three levels: high knowledge score (≥ 66.36%), moderate knowledge score (50% < 66.36%), and low knowledge score (< 50%). The diversity of the scores on the knowledge scale indicates variation in participants’ knowledge of the trueness or falseness of the measuring items of this scale. The item that received the highest percentage of ‘true’ responses was ‘*A primary health-care system is based on the belief that if enough effort is spent developing health-care technology systems, quality of life is enhanced*’ (n = 147; 93.6%). Conversely, the findings revealed that the item that received the lowest percentage of ‘true’ responses was ‘*Acute care services are not considered part of a primary health-care system*’ (n = 29; 18.5%).

**Table 2:** Correct Answers of Students’ Knowledge towards Primary Health Care (N = 157).

Item	True	
	N	(%)
1. Accessibility to health care is a basic concept of primary health care.	120	76.4%
2. The World Health Organization considers primary health care to be the best way to achieve “Health for All.”	133	84.7%
3. The WHO considers that primary health care is equally important for both industrialized and developing countries.	132	84.1%
4. The key approach to achieving primary health care is technology.	67	42.6%
5. Provincial health economists should be the key planners in any primary health care project.	120	76.4%
6. One major emphasis of primary health care is disease prevention.	110	70.0%
7. Within a primary health-care system, safe, adequate drinking water is considered as important as professional health services.	66	42.0%
8. A statement of commitment to primary health care was ratified at the International Conference held at Alma Ata in 1978.	87	55.4%
9. In a primary health-care system, lay health-care personnel replace most health professionals.	126	80.3%
10. Primary health care emphasizes the importance of a biomedical approach to health care.	133	84.7%
11. An increase in physicians is needed in Jordan to fully implement primary health care.	133	84.7%
12. The Jordanian Nurses Association supports the goals of primary health care.	126	80.3%
13. The primary health-care movement began under the auspices of the International Council of Nurses.	66	42.0%
14. Many governmental departments, such as Agriculture and Municipal Planning, are important for the implementation of the goals of primary health care.	66	42.0%
15. Within a primary health-care framework, the health-care system is considered to be the key determinant of the population’s health.	110	70.0%
16. Supporters of primary health care consider that the most effective way to improve the mental health of the community is to increase the number of psychiatrists.	133	84.7%
17. In a primary health-care system, nurses have an increased role in prevention and promotion.	132	84.1%
18. A primary health-care system centralizes the planning and implementation of health care.	110	70.0%
19. A primary health-care system is based on the belief that if enough effort is spent developing health-care technology systems, quality of life is enhanced.	147	93.6%
20. Primary health care involves, among other activities, working on underlying problems that affect social and emotional health.	110	70.0%
21. An example of a primary health-care strategy to improve the health of the community is to increase the number of cardiac specialists.	123	78.3%
22. Rehabilitative services are part of primary health care.	126	80.3%
23. Improved health education is a key concept in primary health care.	133	84.7%
24. Cooperation between governments and voluntary organizations is a key concept in primary health care.	135	83.7%
25. The primary health-care movement was initiated at the International Health Promotion Conference in 1988.	69	44%
26. Primary health-care approaches take into consideration ways to provide culturally appropriate care.	126	80.3%
27. Community participation is central to an effective primary health-care system.	67	42.6%
28. Within the primary health-care perspective, many functions that lay health-care workers currently perform are the responsibility of health-care professionals.	80	51%
29. Acute care services are not considered part of a primary health-care system.	29	18.5%
30. In a primary health-care system, efforts are made to use the least expensive technology and personnel to achieve positive health outcomes.	126	80.3%
31. Primary health care focuses on setting targets and plans of action to meet national health goals.	118	75.2%
32. One of the five key principles of primary health care is the provision of high-quality, episodic medical care.	126	80.3%
33. Primary health-care services do not include curative or palliative care.	132	84.1%
34. Our current provincial health-care system is based on a primary health-care model.	48	30.6%
35. A coalition of seniors, police, and local merchants working to improve neighborhood safety is an appropriate primary health-care strategy.	120	76.4%

N: number, %: Percentage.

Table 3 presents the results of knowledge scores of the total sample of nursing students (n = 108), ranging from 18.5 to 93.6. Measures of central tendency for knowledge scores are mean 68.8, standard deviation 30.22, and mode 80.3.

**Table 3:** Knowledge-related true answers score (N = 108).

Knowledge score	N	X	SD	Min	Max
108	68.8	30.22	18.4	93.6

*Note: N = number; % = percentage; X = mean; SD = standard deviation; Min = minimum; Max = maximum.

**Fig 1:**
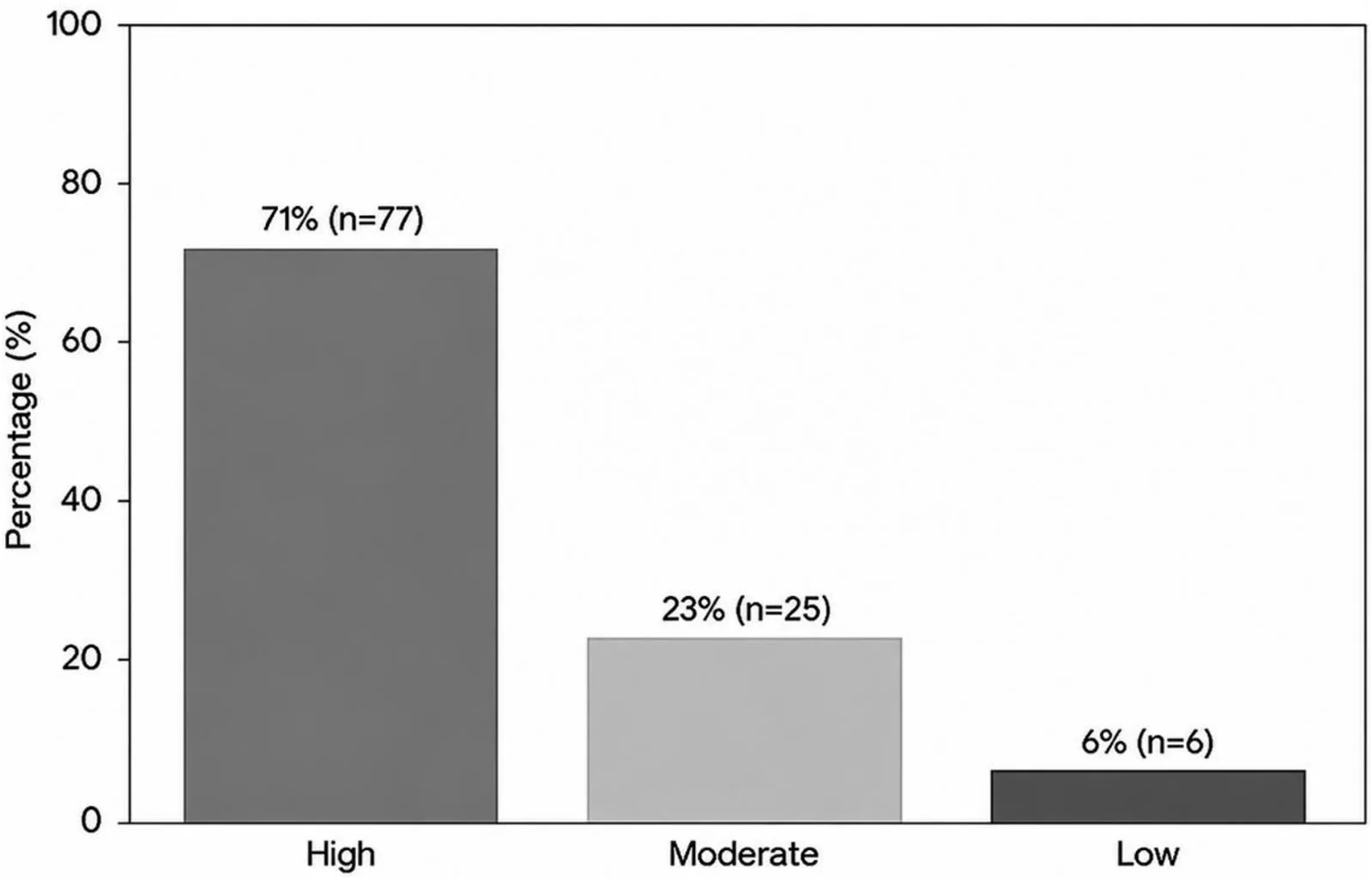
Distribution of Knowledge Levels Toward Primary Health Care (PHC) in Jordan (n = 108). The bar chart illustrates that 71% of participants possessed a high knowledge score, while only 6% fell into the low knowledge category.

Furthermore, the results based on the scores of knowledge that were categorized into three levels (i.e., high, moderate, and low) demonstrated that 71% (n = 77) of the participants had a high knowledge score, 23% (n = 25) had a moderate knowledge score, and 6% (n = 6) had a low knowledge score concerning PHC in Jordan. Fig 1 is a visual representation of the results obtained, which categorize these levels of knowledge (see supporting materials).

### The PHCQ (Attitudes scale)

The distribution of answers to the questionnaire assessing nursing students’ attitudes towards PHC in Jordan is shown in Table 4. For each of the 34 items in the questionnaire, answers are divided into four categories: strongly disagree, disagree, agree, and strongly agree. For items with negative meaning, the study reversed item codes using SPSS and placed a symbol (R) beside them. The scoring system of this scale is categorized according to three levels: highly positive attitudes (≥ 65.91%), moderate attitudes (65.91% - 50%), and less positive attitudes (< 50%). The results showed different scores on the attitude scale, which indicates variation in participants’ attitudes toward their agreement or disagreement with the measuring items of this scale. The results revealed that the item with the highest mean score was ‘*Volunteers and lay personnel could provide many services that are currently provided by health-care professionals*’ (m = 3.71, SD = 0.457). Conversely, the findings showed that the item with the lowest mean score was ‘*More health-care dollars should go towards developing technological equipment to diagnose disease*’ (m = 3.34, SD = 0.572).

**Table 4:** Students’ Attitudes towards Primary Health Care (N = 157).

Item	X	SD
1. More healthcare dollars should go towards developing technological equipment to diagnose disease.	3.34	0.572
2. Increased medical specialization is needed to improve the community’s health.	3.49	0.666
3. All people in a country should have access to basic health care, even if it means that some people would receive fewer services than they currently receive.	3.59	0.631
4. Nurses could provide many health-care services that physicians currently provide.	3.49	0.666
5. Most children need “well child” care from a pediatrician rather than from a general practitioner (i.e., family doctor).	3.30	0.729
6. Fee-for-service as a method of payment for physicians should be discontinued, and another payment system substituted.	3.64	0.481
7. Access to good health care is a fundamental right of all people.	3.73	0.461
8. More money needs to be spent on health promotion and disease prevention, even if this means less money is available for highly specialized treatment and acute care.	3.61	0.489
9. Volunteers and lay personnel could provide many services that are currently provided by healthcare professionals.	3.71	0.457
10. The physician is the best person to keep people well.	3.55	0.499
11. Community members should have input into how healthcare dollars are spent in their communities.	3.52	0.626
12. Many tasks that physicians currently perform could be carried out equally well by nurses.	3.59	0.578
13. Helping people learn to stay well is an important role for nurses.	3.53	0.636
14. Doctors and nurses should spend more time providing information to people about their health situations.	3.53	0.636
15. Childbearing women should have an increased role in decision-making regarding procedures used during their labor and delivery.	3.73	0.461
16. More effort should be taken by healthcare professionals to discourage traditional healers from providing healthcare.	3.53	0.636
17. More money needs to be spent on accident prevention for children and adolescents, even if this means longer waits for elective surgery for some patients.	3.52	0.704
18. The health-care system should take increased responsibility for developing programs to address problems such as family violence.	3.34	0.572
19. The way to solve the problem of high death rates from heart disease is to screen children for high lipid levels.	3.34	0.667
20. Each person has a responsibility to maintain his/her health.	3.59	0.631
21. Trained midwives should be accessible for women who choose to use the services of this health professional.	3.49	0.666
22. As much emphasis should be placed on assisting people to cope with their health problems as is placed on diagnosing and treating them.	3.30	0.729
23. Getting consumers involved in decisions about how health-care dollars are spent would result in lower-quality health care.	3.66	0.515
24. People should have liberal access to health information from doctors and nurses so they can participate fully in decisions affecting their health.	3.64	0.481
25. Provincial governments need to assume more responsibility to ensure that people in all areas of the province have access to needed medical services, even if this means controlling where some physicians can practise.	3.73	0.461
26. Individuals are capable of taking primary responsibility for their own health.	3.61	0.489
27. If individuals can afford to pay user fees for services, they should be expected to do so.	3.71	0.457
28. Primary health care is a useful approach for developing countries, but has little relevance for industrialized countries. (R)	3.55	0.499
29. Currently used health indicators (morbidity and mortality) are useful but limited ways of determining the effectiveness of primary health care.	3.52	0.626
30. Primary health care is appropriate only for low-income groups and communities. (R)	3.59	0.578
31. Cultural variations in health-care practices are over-emphasized.	3.53	0.636
32. Community participation is a deterrent in implementing primary health-care programs. (R)	3.53	0.636
33. No matter where our health-care dollars are spent, we will never be able to reduce the incidence of disease in Jordan. (R)	3.37	0.554
34. All health-care professionals have a role in primary health care.	3.29	0.531

X: mean, SD: Standard deviation.

Table 5 contains the results of attitude scores of the total sample of nursing students (n = 157), ranging from 99 (less positive attitude) to 136 (more positive attitude), with measures of central tendency for attitude scores mean 120.47; standard deviation 8.60.

**Table 5:** Attitudes scores (N = 157).

Attitudes score	N	X	SD	Min	Max
157	120.47	8.60	99	136

*Note: X = mean; SD = standard deviation; Min = minimum; Max = maximum; R = reversed item.

**Fig 2:**
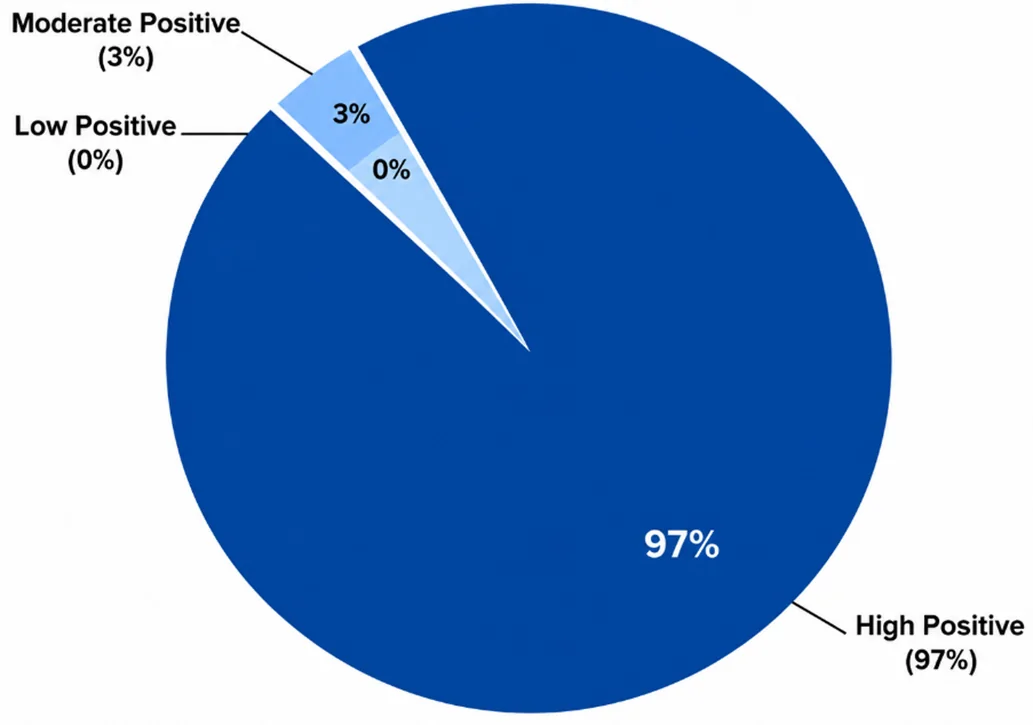
Participants’ Attitude Levels Toward Primary Health Care (PHC) in Jordan (n = 157). The pie chart demonstrates an overwhelmingly positive outlook, with 97% of participants scoring in the high positive attitude range.

Furthermore, the results based on the scores of attitudes were categorized into three levels (high positive attitude, moderate, and low positive attitude), revealing that most of the participants (97%) demonstrated a high positive attitude score toward PHC in Jordan, and that 3% of participants had a moderate positive attitude. Fig 2 is a visual representation of the results gained, which categorize these levels of attitude (see supporting materials).

### The difference between the level of attitude, sociodemographic, and academic-related factors toward PHC

The results presented in Table 6 revealed that there was an association between participants’ gender and their attitudes towards primary health care in Jordan. The attitude scale revealed a significant association between gender (p < 0.05) and the attitude of the participants. The results indicated that males had a higher mean score of positive attitudes towards primary health care (1.54 ± 0.177) than females. Additionally, the attitude scale revealed a significant association between age (p < 0.05) and the attitude of the participants. The group of participants aged 26–30 had the highest score of attitudes towards primary health care (1.29 ± 0.188) compared with other age groups. The attitude scale showed a significant association between monthly income (p < 0.05) and the attitude of the participants. The mean score of attitudes towards primary health care (1.30 ± 0.433) of participants with high monthly income (751–1000 JOD) was greater than that of participants with lower monthly income.

**Table 6:** Attitude, Socio-Demographics, and Academic Factors Association.

Variable		Frequency	Mean+SD	P-value
Age	20-25 years	117	1.18 + 0.227	0.000**^b^
	25 < -30 years	33	1.29 + 0.188	
	Above 30 years	7	1.20 + 0.214	
Monthly income	250-500 JOD	61	1.09 + 0.190	0.003**^b^
	501-750 JOD	60	1.22 + 0.222	
	751-1000 JOD	29	1.30 + 0.433	
	Above 1000 JOD	7	1.17 + 0.155	
Academic performance (GPA)	Faire	24	1.22 + 0.153	0.000**^b^
	Good	73	1.12 + 0.154	
	Very good	43	1.16 + 0.232	
	Excellent	17	1.43 + 0.155	
Number of Completed Credits	60-80 hours	13	1.16 + 0.166	0.001**^b^
	81-100 hours	60	1.23 + 0.136	
	101-120 hours	77	1.14 + 0.263	
	Above 120 hours	7	1.11 + 0.065	
Hours Spent Studying per Week	<10 hours	25	1.17 + 0.071	0.007**^b^
	10-20 hours	102	1.23 + 0.255	
	21-30 hours	30	1.25 + 0.163	
Gender	Male	90	1.54 + 0.177	0.000**
	Female	67	1.53 + 0.161	
University type	Governmental	55	1.15 + 0.143	0.735^a^
	Private	102	1.07 + 0.173	
Intent to leave nursing in the future	Yes	34	1.11 + 0.199	0.000**^a^
	No	123	1.18 + 0.121	

* Significance level P value >0.05 ** significance level P value.0.01 > a = t-test; b = ANOVA.

Moreover, the attitude scale showed a significant association between intent to leave nursing in the future (p < 0.05) and the attitude of the participants. The results indicated that those who have no intent to leave nursing in the future had a higher mean score of attitudes towards primary health care (1.18 ± 0.121) than those who have the intent to leave nursing in the future. For academic-related factors, the results showed that students with excellent GPAs had the highest mean score of attitudes (1.43 ± 0.155) compared to those with low academic performance. The attitude scale showed a significant association between academic performance (p < 0.05) and the attitudes of the participants. Furthermore, the results demonstrated that students who completed a number of credits (81–100 hours) had the highest mean score of attitudes (1.23 ± 0.136) compared to those with a lower number of credits. The attitude scale showed a significant association between the number of completed credits (p < 0.05) and the attitude of the participants. Moreover, the results showed that students with a high number of study hours per week (21–30 hours) had the highest mean score of attitudes (1.25 ± 0.136) compared to those with a low number of study hours per week (<10 hours). The attitude scale showed a significant association between hours spent studying per week (p < 0.05) and the attitude of the participants.

### Predictors of attitudes towards PHC among undergraduate nursing students

To address this question, the study performed multiple regression analysis to achieve its aim. Based on Table 7, gender (B = 0.173), academic performance (B = 0.044), and intent to leave nursing in the future (B = 0.122) were significant predictors of nursing student attitudes toward PHC. The obtained unstandardized B coefficients have associated probability values of 0.000 each, which is significant at a < 0.05 significance level. Therefore, gender, academic performance, and intent to leave nursing in the future were significant predictors of undergraduate nursing student attitudes toward PHC in Jordan.

**Table 7:** Multiple Regressions to Predict Factors of Students’ PHC Attitudes.

	Unstandardized coefficients		Standardized coefficients		95% CI LL UL	
**Model**	**B**	**Std. error**	**Beta**	**T**		**Sig.**
Gender	0.173	0.036	-0.120	4.925	0.102-0.244	0.000**
Age	0.032	0.033	-0.014	0.969	-0.033-0.097	0.675
Monthly income	0.057	0.055	.432	1.036	-0.051- 0.165	0.123
Academic performance (GPA)	0.044	0.016	0.245	2.750	0.013- 0.075	0.002**
Hours Spent Studying per Week	0.034	0.026	-0.56	1.307	-0.017- 0.085	0.342
Number of Completed Credits	0.040	0.031	0.388	1.290	-0.021- 0.101	0.087
Intent to leave nursing in the future	0.122	0.051	0.041	2.392	0.022- 0.222	0.000**

* Significance level P value 0.05 > ** significance level P value. < 0.01.

**Note: university type was excluded from the regression equation as it was not correlated.**

The results of the model summary in Table 8 provide details concerning the characteristics of the model. In the current case, several predictors were considered as the main variables. In the regression analysis, demographic characteristics significantly predicted attitudes toward PHC services; however, the cross-sectional design does not permit causal interpretation. R-value was 0.672, which is considered to be good. Furthermore, R-squared shows how the total variation in the dependent variable could be explained by the independent variables. It represents the effectiveness of the model; the value was 0.451, which is good.

**Table 8:** Model Summary.

Model		Sum of Squares	Df	R	R Square
1	Regression	0.295	9	0.672	0.451
	Residual	9.706	147		
	Total	10.001	156		

Df: Degree of freedom.

## Discussion

### The level of knowledge regarding PHC among undergraduate nursing students

Primarily, this study was designed to assess the level of knowledge regarding PHC among undergraduate nursing students in Jordan via the use of the PHCQ. According to the results of this study, undergraduate nursing students exhibit a good understanding of primary healthcare and demonstrate generally positive attitudes toward PHC. Additionally, the results revealed that the majority of undergraduate nursing students displayed a high level of knowledge, which can be attributed to the information they acquired following the introduction of PHC concepts during their third year.

The research findings align with a study conducted by Abu-Baker et al. [6] and the findings of several international studies. For example, Bloomfield et al. [15] reported comparable results associated with a robust healthcare system, which is essential and should be built on the foundation of PHC. Furthermore, the results indicated that nursing programs must graduate competent nurses who can provide people and the community with integrated health care services that incorporate both preventative and curative elements. To improve overall community health, nursing students should be taught the knowledge and abilities required to manage health services by providing them with a robust scientific background and opportunities to conduct research and create plans. According to the Jordanian Nursing Council (JNC) (2016), community nursing is a significant component of training courses; therefore, as a primary domain, it should be integrated into the nursing curriculum in Jordan [30]. Its inclusion may encourage nursing programs in Jordan to integrate PHC into the curriculum both theoretically and clinically.

Furthermore, this study’s results are consistent with the work of Ukoha & Dube [7], which also addressed the PHC nursing students’ knowledge and attitudes towards the provision of preconception care. Their research revealed that although students were informed and had a positive attitude toward PHC, they were disadvantaged by the lack of PHC resources in many practices. Therefore, to facilitate the correct implementation of PHC, it is recommended that health care workers be provided with the resources they require. This may indicate that curriculum development and educational preparation are crucial factors in determining the scope of student knowledge concerning PHC. Public health courses, community health nursing, and hands-on fieldwork are examples of programs that incorporate PHC principles and provide students with a thorough understanding. Students enrolled in courses with little exposure to PHC, as in Khattak et al. [10], may not possess the required practical knowledge and competencies. Prioritizing PHC in the curriculum is essential for ensuring that educational institutions develop well-rounded healthcare professionals.

### The levels of attitudes towards PHC among undergraduate nursing students

The findings of this study concerning the levels of attitudes towards PHC revealed that nursing students typically display positive attitudes about a range of healthcare-related topics, including PHC. Despite these positive attitudes, participants in this study measured attitudes toward the volunteers and lay personnel who could provide many services that are currently provided by healthcare professionals. This result aligns with regional and international studies, including those presented by Bloomfield et al. (2015), who noted that students with exposure to PHC settings tend to develop a favorable attitude toward community-based care. Additionally, [31] demonstrated that total knowledge ranged from 19.68 to 95.78, with the mean knowledge score being 69.19, and total attitude scores ranged from 33.12 to 93.88, with a mean score of 70.45, which suggests a fundamental appreciation for healthcare practices that could be used to advance PHC principles. Despite the lack of positive sentiment, there is variation regarding the healthcare budget that should be allocated to developing technological equipment for disease diagnosis.

The findings also asserted the role of the nursing curriculum in determining how students perceive PHC. PHC principles, community health nursing, and public health courses (theoretical & clinical) can all be incorporated to improve students’ attitudes. PHC should be given priority in curriculum development to foster positive attitudes; however, one study indicates that limited exposure to PHC settings during training may lead to less favorable attitudes [10]. Additionally, the effects on students’ attitudes can be shaped by cultural and social contexts: for example, Khattak et al. [10] reported more variable attitudes, particularly in settings where PHC is less emphasized or perceived as less prestigious than hospital-based care. This contrast suggests that attitudes toward PHC are influenced by a range of factors, including health system priorities and personal values.

Overall, Nursing students demonstrated generally positive attitudes toward primary healthcare services, and this is also true of PHC services. But there is variation because of cultural, experiential, and educational factors. To address this, nursing education programs should incorporate comprehensive PHC content and offer hands-on experience to promote consistently positive attitudes toward PHC. Based on the aforementioned points, it is evident that such attitudes and the creation of focused interventions require additional study. The current research noted that most of the participants agreed that healthcare practitioners should spend more time providing medical information to their patients.

### The associations between the level of knowledge, attitudes, sociodemographic factors, and academic-related factors

The results of this research revealed a significant association between student knowledge, attitudes, demographics, and academically related factors toward PHC. The results found statistically significant >0.05 for the knowledge and attitude levels, which asserts the importance of understanding how students’ knowledge, attitudes, sociodemographic, and academic-related factors relate to PHC, thereby enhancing teaching methods and creating qualified nursing professionals. This study explores these associations, highlighting relevant academic perspectives and empirical evidence. The findings showed a robust association between students’ attitudes and their knowledge about PHC, indicating that higher knowledge levels tend to be linked with more positive attitudes. This association is consistent with previous studies indicating that knowledge and attitudes toward PHC are interrelated constructs that often develop concurrently during professional education [1,32].

Additionally, this research revealed that demographic characteristics were significantly associated with PHC attitudes and knowledge, with demographic parameters such as age, gender, socioeconomic level, and academic achievement influencing students’ attitudes and knowledge of PHC. For example, older students or those with prior healthcare experience may possess a better understanding and display more positive attitudes [6,33]. Gender may also play a role: some studies suggest that female students are more likely to have a positive opinion of PHC (perhaps due to their greater empathy); however, this finding should be treated with caution due to its context-dependent nature [34]. Contrastingly, this study reports that males tend to have a more positive attitude than females, which may reflect contextual factors (such as exposure to PHC clinical settings, educational experience, and/or career expectations). However, it should be noted that these results may be influenced by sociocultural and environmental factors rather than inherent characteristics [34,35].

Academic variables impact PHC knowledge and attitudes: GPA and the number of credits earned are two examples of academic elements that have a significant impact on students’ knowledge and attitudes about PHC. Typically, students who participate in PHC-focused programs that combine community health nursing, public health, and hands-on fieldwork graduate with higher levels of knowledge and more positive attitudes. Conversely, inadequate exposure to PHC topics in the curriculum may lead to negative attitudes and knowledge, as well as poor comprehension. Previous research suggests that academic progression and performance are positively correlated with students’ competency and preparedness in PHC-related domains [36,37]

According to the findings of this study, knowledge, attitudes, sociodemographic, and academic characteristics interact and have a dynamic, interrelated relationship. For example, a positive attitude regarding PHC may encourage students to seek out additional information, and a supportive learning environment may enhance attitudes and knowledge. Demographic characteristics may also be associated with how students react to instructional tactics: younger students may require more basic information, while older students might benefit from exposure to more complex PHC concepts. Furthermore, the interaction between students’ academic traits, attitudes, knowledge, and demographics concerning PHC is complex and multifaceted. According to Calma et al. [9], three themes influence curricula: students’ intention to work in primary healthcare, their knowledge, and their attitudes toward primary healthcare; therefore, to ensure that students receive a comprehensive PHC education that fosters knowledge and positive attitudes, educational institutions must consider these factors when developing curricula. However, given the cross-sectional nature of the study, no causal inferences can be made, and these relationships should be interpreted as correlational rather than causal.

### Factors that predict the level of attitudes towards PHC among undergraduate nursing students

This study comprised several elements that identified the factors predicting the level of attitudes towards PHC. The results concerning demographics and academic performance factors (such as gender, GPA, and intent to leave nursing in the future) have been found to significantly impact students’ attitudes towards PHC (p > 0. 05). The regression analysis revealed that students’ attitudes towards PHC was predicted by gender, academic performance (GPA), and intent to leave nursing in the future, each of which are acknowledged as crucial components of PHC. The students’ age, GPA, and intention to leave nursing provide the most support. Additionally, these factors become even more important during PHC education and practices, and notably, when assessing students’ attitude levels, they become increasingly significant.

Previous studies [13] reported similar findings, i.e., that attitudes toward PHC can be predicted by demographic factors (such as age and gender). For example, students who are older or have prior healthcare experience might display a deeper understanding of PHC principles. Gender differences may also exist: some research indicates that female students are more likely to have positive attitudes because they are more empathetic [13]. The underlying reasons for selecting a career in nursing motivate students’ perceptions of PHC, which, in turn, may be influenced by their reasons for wanting to become nurses. While people who are driven primarily by material or status-related considerations might have less positive attitudes, those who are driven by a desire to serve the community might be more open to PHC principles.

## Conclusion

This study focused on assessing Jordanian nursing students’ knowledge and attitudes towards PHC. The study identified several significant outcomes concerning students’ knowledge and attitudes toward PHC. Regarding knowledge, it was noted that the majority of the students expressed high knowledge in primary care, which clarifies the significance of a biomedical approach to healthcare. Regarding attitude, most of the participants demonstrated a highly positive attitude towards PHC. This study established that gender, academic performance (GPA), and intention to leave nursing in the future had a significant correlation with participants’ knowledge and attitudes. According to the study’s findings, knowledge, attitudes, sociodemographic, and academic characteristics interact and have a dynamic, interrelated relationship. For example, a positive attitude regarding PHC may encourage students to seek out additional information, and a supportive learning environment may enhance their existing attitudes and knowledge. Additionally, the findings asserted the role of the nursing curriculum in influencing how students perceive PHC; therefore, PHC principles, community health nursing, and public health courses (theoretical & clinical) should be incorporated into the curriculum to enhance students’ attitudes. PHC should be given priority in curriculum development to foster positive attitudes; however, one study indicates that limited exposure to PHC settings during training may lead to less favorable attitudes. Additionally, the effects on students’ attitudes are shaped by cultural and social contexts. Overall, Jordanian undergraduate nursing students have positive attitudes about medical services provided by PHC, and this is also true of PHC; however, some variations can be attributed to a range of cultural, experiential, and educational factors. Programs for nursing education should incorporate comprehensive PHC content and offer hands-on experience to promote consistently positive attitudes toward PHC.

### Implications

The results regarding students’ knowledge and attitudes can facilitate the creation of all-encompassing educational frameworks that prioritize community service, health promotion, and PHC principles. Furthermore, the development of conceptual models and studies concerning students’ attitudes and knowledge about PHC can enable the development of conceptual models that describe how exposure to education, knowledge acquisition, attitude formation, and healthcare practice are related.

According to the study’s findings, nursing educators must receive comprehensive PHC training. Such training will enhance patient education and foster increasingly positive attitudes toward PHC. Additionally, these findings may improve clinical practice via the introduction of additional PHC-focused clinical placements, thereby providing students with hands-on experience in community settings. When considering student assessment and evaluation, the current findings can help develop tools to measure nursing students’ attitudes and knowledge concerning PHC, thereby enabling educators to identify areas for improvement. By emphasizing the importance of PHC in nursing education and advocating for government funding of PHC-centered training programs, the findings of the current study can guide policymaking and help establish ongoing education initiatives for nursing professionals and students, ensuring they stay up to date with PHC principles and procedures.

By identifying the influential factors, the theoretical research can assist in determining the academic, cultural, and demographic elements that affect nursing students’ perceptions of PHC in Jordan. Future research and curriculum design can be guided by these insights: analyzing the knowledge-attitude-behavior link (via the theories of behavior change in health education) can provide the basis for theoretical research concerning how nursing students’ understanding of PHC results in positive attitudes and behaviors.

### Strengths & limitations

Given the transition toward PHC as a key tactic for attaining universal health coverage (UHC), this research contains several strengths. Its emphasis on PHC aligns with global health goals: nursing students are the PHC’s future workforce; therefore, evaluating their knowledge and attitudes is essential because their perception may have a direct impact on the standard and focus of care. Additionally, contemporary research concerning PHC education and attitudes in Middle Eastern countries is scarce; therefore, this research addresses a significant knowledge gap in this area. The results might help curriculum developers, nursing educators, and health policy makers better prepare students for PHC responsibilities. The outcomes of this study provide crucial information concerning the situation of nursing students and their knowledge and attitudes toward PHC in Jordan. Moreover, the current study provides valuable research insights concerning this domain and attempts to add further discussions and evidence, all of which could enhance the knowledge and attitudes toward PHC in Jordan.

As with any research project, the results of this study should be accompanied by an acknowledgement of its limitations. Despite the cross-sectional nature of this study, the researcher concentrated on four private and government universities in Jordan, thereby capturing data at a single point in time. This approach limits the ability to assess changes in students’ knowledge and attitudes over time, preventing a comprehensive understanding of how these factors evolve throughout their education. The use of convenience sampling and recruitment from universities in central Jordan may limit the representativeness of the sample; therefore, the findings may not be fully generalizable to all nursing students in Jordan. Furthermore, PHCQ may not fully capture the contemporary scope of PHC, particularly those factors related to technological advancements; however, the PHCQ is acknowledged as a suitable approach for evaluating nursing students’ baseline knowledge and attitudes concerning conventional PHC topics. Additionally, it should be noted that when evaluating the knowledge results, it is accepted that the questionnaire design, specifically the preponderance of positively framed items, may have an impact on response patterns. However, because it contains both knowledge-based items (with correct answers) and attitudinal items (without definitive answers), the PHCQ acts as a composite measure rather than a strictly objective test.

### Recommendations

The results of this study demonstrate that nursing students’ attitudes and knowledge concerning PHC vary, and there are important correlations with academic and demographic factors. Based on its findings, this research makes the following recommendations:

- Curriculum alignment with PHC competencies: Given the reported difference in knowledge levels, the nursing curriculum should be assessed to ensure that fundamental PHC ideas are consistently and sufficiently covered at all academic levels.
- Early and progressive integration of PHC content: Knowledge and attitudes were linked to academic advancement; therefore, rather than being limited to certain courses, PHC-related content should be introduced early and reinforced throughout the duration of the program.
- Targeted academic support: Academic performance (such as GPA) was linked to PHC knowledge and attitudes; therefore, students with inferior academic achievement may benefit from additional academic support or reinforcement techniques.
- Contextual adaptation of measurement instruments: Future research should consider updating or modifying measurement tools to better reflect modern PHC principles and guarantee balanced item design, considering the PHCQ’s constraints.
- Additional research employing complementary methodologies: This research was cross-sectional and quantitative in nature; therefore, future research could utilize qualitative methodologies to better understand students’ experiences and views of PHC and to explain the observed differences in attitudes and knowledge. Moreover, future studies using probability sampling methods and broader geographic coverage across Jordan would strengthen representativeness.

## Supporting information

S1 Text Arabic version of the tool. The Arabic translation of the Primary Health Care Questionnaire (PHCQ).
(DOC)
