# Supplementary material for: Undergraduate nursing students’ knowledge and attitudes towards Primary Health Care
Source: PLOS Glob Public Health. 2026 Sep 1;6(9):e0007178. doi: 10.1371/journal.pgph.0007178 (PMC13533354; doi:10.1371/journal.pgph.0007178)
Supplement: Supplementary file 1 [file pgph.0007178.s004.docx]

Responses to reviewers

| Reviewer comment | Response |
| --- | --- |
| Journal Requirements:  If the reviewer comments include a recommendation to cite specific previously published works, please review and evaluate these publications to determine whether they are relevant and should be cited. There is no requirement to cite these works unless the editor has indicated otherwise. | Thank you for your comments  Done |
| Please review your reference list to ensure that it is complete and correct. If you have cited papers that have been retracted, please include the rationale for doing so in the manuscript text, or remove these references and replace them with relevant current references. Any changes to the reference list should be mentioned in the rebuttal letter that accompanies your revised manuscript. If you need to cite a retracted article, indicate the article’s retracted status in the References list and also include a citation and full reference for the retraction notice. | Done |
| Reviewer #1: The paper still requires a careful and comprehensive language edit. | Language editing was done |
| Figure Resubmissions:    While revising your submission, we strongly recommend that you use PLOS’s NAAS tool (https://ngplosjournals.pagemajik.ai/artanalysis) to test your figure files. NAAS can convert your figure files to the TIFF file type and meet basic requirements (such as print size, resolution), or provide you with a report on issues that do not meet our requirements and that NAAS cannot fix. | Done and attached |
|  |  |
