# Supplementary material for: Undergraduate nursing students’ knowledge and attitudes towards Primary Health Care
Source: PLOS Glob Public Health. 2026 Sep 1;6(9):e0007178. doi: 10.1371/journal.pgph.0007178 (PMC13533354; doi:10.1371/journal.pgph.0007178)
Supplement: Supplementary file 2 [file pgph.0007178.s005.docx]

Responses to reviewers

| Comment | Response | modifications |
| --- | --- | --- |
| Please review your reference list to ensure that it is complete and correct. If you have cited papers that have been retracted, please include the rationale for doing so in the manuscript text, or remove these references and replace them with relevant current references. Any changes to the reference list should be mentioned in the rebuttal letter that accompanies your revised manuscript. If you need to cite a retracted article, indicate the article’s retracted status in the References list and also include a citation and full reference for the retraction notice | Thank you for your comment | Done |
| Does this manuscript meet PLOS Global Public Health’s publication criteria? Is the manuscript technically sound, and do the data support the conclusions? The manuscript must describe methodologically and ethically rigorous research with conclusions that are appropriately drawn based on the data presented | Thank you for your comment | We tried our best to follow the journal guideline accurately and connect our results and conclusion to gather  The conclusion was reviewed and modified |
| PLOS Global Public Health does not copyedit accepted manuscripts, so the language in submitted articles must be clear, correct, and unambiguous. Any typographical or grammatical errors should be corrected at revision, so please note any specific errors here. | Thank you for your comment | Proofreading and editing services were utilized by a professional company, and modifications were made |
| Reviewer #1: I'm sorry to say that I still do not think this manuscript is ready to be accepted and published. The minimal changes made do not address many of the inconsistencies in the manuscript's language. There are problems with inconsistent tenses, misused transition words, and word choice. These challenges make many of the paper's findings difficult to understand and assess for validity. | Thank you for your careful review and for highlighting the remaining concerns regarding the language quality of our manuscript.  In response to your comments, we have undertaken a comprehensive language revision of the entire manuscript. To ensure the highest standard of academic English, the manuscript was professionally proofread and edited by a specialist with expertise in scientific and academic writing. During this process, particular attention was given to correcting inconsistencies in verb tenses, improving the use of transition words, refining word choice, and enhancing the overall clarity, coherence, and readability of the manuscript.  We sincerely appreciate your constructive feedback, which has helped us substantially improve the quality and comprehensibility of our work. We hope that the revised manuscript now addresses your concerns and is suitable for publication. |  |
