# Supplementary material for: Undergraduate nursing students’ knowledge and attitudes towards Primary Health Care
Source: PLOS Glob Public Health. 2026 Sep 1;6(9):e0007178. doi: 10.1371/journal.pgph.0007178 (PMC13533354; doi:10.1371/journal.pgph.0007178)
Supplement: Supplementary file 4 [file pgph.0007178.s007.docx]

Responses for reviewers

| **comment** | **response** | **modifications** |
| --- | --- | --- |
| 1. If the authors have adequately addressed your comments raised in a previous round of review and you feel that this manuscript is now acceptable for publication, you may indicate that here to bypass the “Comments to the Author” section, enter your conflict of interest statement in the “Confidential to Editor” section, and submit your "Accept" recommendation.  Reviewer #1: (No Response) | - | - |
| 2. Does this manuscript meet PLOS Global Public Health’s publication criteria? Is the manuscript technically sound, and do the data support the conclusions? The manuscript must describe methodologically and ethically rigorous research with conclusions that are appropriately drawn based on the data presented.  Reviewer #1: Partly | Thank you. We acknowledge your evaluation that the manuscript partly meets the publication criteria. In response, we have carefully revised the manuscript to address the concerns related to the clarity of the study’s central message, the consistency between the study objectives, statistical findings, and conclusions, and the appropriate interpretation of the findings based on the cross-sectional study design. |  |
| 3. Has the statistical analysis been performed appropriately and rigorously?  Reviewer #1: I don't know | have carefully re-examined the statistical analysis and revised the Methods and Results sections to provide clearer information regarding the statistical procedures used, the rationale for selecting each test, and the assumptions underlying the analyses. We have also reviewed the consistency between the statistical tests, reported results, tables, and interpretation of the findings. |  |
| 4. Have the authors made all data underlying the findings in their manuscript fully available (please refer to the Data Availability Statement at the start of the manuscript PDF file)?  The PLOS Data policy requires authors to make all data underlying the findings described in their manuscript fully available without restriction, with rare exception. The data should be provided as part of the manuscript or its supporting information, or deposited to a public repository. For example, in addition to summary statistics, the data points behind means, medians and variance measures should be available. If there are restrictions on publicly sharing data—e.g. participant privacy or use of data from a third party—those must be specified.  Reviewer #1: Yes | Thank you |  |
| 5. Is the manuscript presented in an intelligible fashion and written in standard English?  PLOS Global Public Health does not copyedit accepted manuscripts, so the language in submitted articles must be clear, correct, and unambiguous. Any typographical or grammatical errors should be corrected at revision, so please note any specific errors here.  Reviewer #1: Yes |  |  |
| Reviewer 1 | | |
| This writing in this paper is improved. However, its messages are still not clear enough to merit publication. I think it tries to combine too many topics, becoming wordy and lacking clarity on messaging. | Thank you for carefully evaluating our revised manuscript and for recognizing the improvements made in the writing. We appreciate your continued concerns regarding the clarity, focus, and consistency of the manuscript. In response, we have undertaken a comprehensive revision to streamline the manuscript, clarify its central message, remove unnecessary or repetitive content, ensure consistent terminology, and align the Abstract, Results, Discussion, and Conclusions with the actual findings of the study. | Please see highlighted parts |
| Just a few examples:  - Lines 84-86: it cites nurses as "advocates of the nursing discipline" but then goes on to talk about nurses as advocating "for community health". These are very different endeavors. Which is being addressed here?  - Lines 109 and 157 cite the same statistic (but only once does it clarify that this study was with Australian nursing students).  - Lines 501/505: are these predictors or associations?  - Line 534 There is a disconnected phrase " Before gathering data from 157 students enrolled in the nursing major."  - Lines 576-580 and 674-5 say that students exhibited positive attitudes concerning medical services in PHC and PHC services. What is the difference?  I also do not think that the conclusions of the Abstract match the paper's findings and conclusions. | We agree that the previous version attempted to address too many related issues and that this reduced the clarity of the manuscript’s central message. Accordingly, we have revised the manuscript to focus clearly on the assessment of nursing students’ knowledge and attitudes toward primary healthcare services and the examination of demographic factors associated with these outcomes. We have removed or substantially shortened background material that was not directly relevant to the study objectives and revised the interpretation of the findings to clearly distinguish between descriptive findings, associations, and regression-based predictors. We have also carefully reviewed the Abstract and revised the conclusions so that they accurately reflect the findings reported in the Results and Discussion sections. | Please see highlighted lines   - Lines 84-86: We removed the ambiguous reference to nurses as “advocates of the nursing discipline” and revised the relevant section to focus specifically on the contribution of nurses to community health and primary healthcare. - Lines 109 and 157: The repeated statistic did not add value and could create ambiguity regarding the population to which the finding applies. We have removed the duplicated statistic and retained the information only once. We have also clearly identified the population studied in the cited research as Australian nursing students.   The repeated statement should be deleted from the second location.   - Lines 501/505: We agree that the terminology should be consistent with the study design and statistical analyses. Because this is a cross-sectional study, the findings should not be interpreted as demonstrating causality.   We have therefore reviewed the Results, Discussion, and Conclusion sections and revised the terminology to distinguish between:  Associations: findings based on bivariate analyses, such as chi-square tests or correlations. Predictors: variables identified as statistically significant in a regression model, while acknowledging that the cross-sectional design does not establish causation.  We have avoided causal language such as “caused,” “led to,” or “resulted in.”   - Line 534 : the sentence has been revised and integrated into the description of the ethical approval and data collection procedures.   The isolated phrase should be deleted.   - Lines 576-580 and 674-5 : thank you for identifying this terminological ambiguity. We agree that the phrases “medical services in PHC” and “PHC services” may appear to refer to the same concept. We have therefore standardized the terminology throughout the manuscript.   The study consistently refers to students’ attitudes toward primary healthcare services. We have replaced inconsistent expressions such as “medical services in PHC” and “PHC medical services” with the single term “primary healthcare services”, unless a specific distinction is intended by the instrument or source being cited.  We have revised the Abstract conclusion to ensure that it directly reflects the findings presented in the Results section and is consistent with the revised Discussion and Conclusion. The revised conclusion avoids introducing claims that were not directly supported by the study findings and avoids causal interpretations. |
