# Supplementary material for: Undergraduate nursing students’ knowledge and attitudes towards Primary Health Care
Source: PLOS Glob Public Health. 2026 Sep 1;6(9):e0007178. doi: 10.1371/journal.pgph.0007178 (PMC13533354; doi:10.1371/journal.pgph.0007178)
Supplement: Supplementary file 5 [file pgph.0007178.s001.doc]

**النسخة العربية من استبيان الدراسة**

**المعلومات الاجتماعية والديموغرافية**

يرجى الإجابة على الأسئلة التالية بأفضل طريقة ممكنة. بالنسبة للمتغيرات المستمرة، يرجى كتابة الرقم أو القيمة المناسبة في المساحة المخصصة. أما بالنسبة للمتغيرات التصنيفية، يرجى اختيار الإجابة التي تنطبق عليك. ستظل إجاباتك سرية وستُستخدم لأغراض البحث فقط

العمر: 1. 20-25 سنة	2. 26-30 سنة	3. فوق 30 سنة

الدخل الشهري للأسرة: 1. 250-500 دينار	2. 501-750 دينار	3. 751-1000 دينار	4. فوق 1000 دينار

الأداء الأكاديمي (المعدل التراكمي) : 1. مقبول	2. جيد	3. جيد جدا	4. ممتاز
عدد الساعات المعتمدة التي تم إنجازها : 1. 60-80 ساعة	2. 81-100 ساعة	3. 101-120 ساعة	4. فوق 120 ساعة

متوسط عدد ساعات الدراسة في الأسبوع: 1. أقل من 10 ساعات	2. 10-20 ساعة	3. 21-30 ساعة

الجنس: ذكر / أنثى

نوع الجامعة : خاصة / حكومية

هل تنوي ترك مهنة التمريض في المستقبل؟
نعم / لا

**الرعاية الصحية الأولية**

شكرًا لمشاركتك في هذه الدراسة
يهدف استبيان الرعاية الصحية الأولية إلى تقييم معرفتك ومواقفك تجاه الرعاية الصحية الأولية. يتكون هذا الاستبيان من قسمين: مقياس المعرفة ومقياس المواقف. يرجى قراءة التعليمات لكل قسم بعناية والإجابة على كل سؤال بصدق. ستظل إجاباتك سرية

**مقياس المعرفة**
التعليمات
يتضمن هذا القسم 35 عبارة صحيحة أو خاطئة حول الرعاية الصحية الأولية. يرجى قراءة كل عبارة بعناية وتحديد ما إذا كنت تعتقد أنها صحيحة أو خاطئة. لا يوجد حد زمني للإجابة، لكن يُرجى التأكد من الإجابة على جميع العبارات.

نظام التصحيح
الإجابة الصحيحة تمنح نقطة واحدة
الإجابة الخاطئة أو الإجابة المفقودة تمنح صفر نقطة
إجمالي نطاق الدرجات من صفر إلى 35

العبارات

| **خطأ** | **صح** | **مقياس المعرفه** | **الرقم** |
| --- | --- | --- | --- |
|  |  | إمكانية الوصول إلى الرعاية الصحية هي مفهوم أساسي للرعاية الصحية الأولية | 1 |
|  |  | تعتبر منظمة الصحة العالمية أن الرعاية الصحية الأولية هي أفضل وسيلة لتحقيق "الصحة للجميع | 2 |
|  |  | تعتبر منظمة الصحة العالمية أن الرعاية الصحية الأولية مهمة بنفس القدر لكل من البلدان الصناعية والنامية | 3 |
|  |  | النهج الأساسي لتحقيق الرعاية الصحية الأولية هو التكنولوجيا | 4 |
|  |  | يجب أن يكون الاقتصاديون الصحيون الإقليميون هم المخططون الأساسيون لأي مشروع للرعاية الصحية الأولية | 5 |
|  |  | إحدى النقاط الرئيسية للرعاية الصحية الأولية هي الوقاية من الأمراض | 6 |
|  |  | ضمن نظام الرعاية الصحية الأولية، يعتبر توفير مياه شرب آمنة وكافية بنفس أهمية الخدمات الصحية المهنية | 7 |
|  |  | تم التصديق على بيان الالتزام بالرعاية الصحية الأولية في المؤتمر الدولي الذي عقد في ألما آتا عام 1978 | 8 |
|  |  | في نظام الرعاية الصحية الأولية، يحل العاملون في الرعاية الصحية العامة محل معظم المهنيين الصحيين | 9 |
|  |  | تركز الرعاية الصحية الأولية على أهمية النهج الطبي الحيوي في الرعاية الصحية | 10 |
|  |  | زيادة عدد الأطباء ضرورية في كندا لتنفيذ الرعاية الصحية الأولية بشكل كامل | 11 |
|  |  | تدعم جمعية الممرضات الكندية أهداف الرعاية الصحية الأولية | 12 |
|  |  | بدأت حركة الرعاية الصحية الأولية تحت رعاية المجلس الدولي للتمريض | 13 |
|  |  | العديد من الإدارات الحكومية، مثل الزراعة والتخطيط البلدي، مهمة لتنفيذ أهداف الرعاية الصحية الأولية | 14 |
|  |  | ضمن إطار عمل الرعاية الصحية الأولية، يعتبر النظام الصحي هو العامل الرئيسي الذي يحدد صحة السكان | 15 |
|  |  | يدعم مؤيدو الرعاية الصحية الأولية أن الطريقة الأكثر فعالية لتحسين الصحة النفسية للمجتمع هي زيادة عدد الأطباء النفسيين | 16 |
|  |  | في نظام الرعاية الصحية الأولية، للممرضات دور متزايد في الوقاية والترويج | 17 |
|  |  | نظام الرعاية الصحية الأولية يركز على تخطيط وتنفيذ الرعاية الصحية بشكل مركزي | 18 |
|  |  | نظام الرعاية الصحية الأولية يعتمد على الاعتقاد بأن الجهود المبذولة لتطوير نظم التكنولوجيا الصحية تعزز جودة الحياة | 19 |
|  |  | تشمل الرعاية الصحية الأولية، إلى جانب أنشطة أخرى، العمل على المشاكل الأساسية التي تؤثر على الصحة الاجتماعية والعاطفية | 20 |
|  |  | كمثال على استراتيجية الرعاية الصحية الأولية لتحسين صحة المجتمع هو زيادة عدد أطباء القلب | 21 |
|  |  | تشمل خدمات الرعاية الصحية الأولية الخدمات التأهيلية | 22 |
|  |  | تحسين التثقيف الصحي هو مفهوم رئيسي في الرعاية الصحية الأولية | 23 |
|  |  | التعاون بين الحكومات والمنظمات التطوعية هو مفهوم رئيسي في الرعاية الصحية الأولية | 24 |
|  |  | بدأت حركة الرعاية الصحية الأولية في المؤتمر الدولي لتعزيز الصحة عام 1988 | 25 |
|  |  | تأخذ نهج الرعاية الصحية الأولية في الاعتبار الطرق لتوفير رعاية مناسبة ثقافيًا | 26 |
|  |  | المشاركة المجتمعية هي محور رئيسي في نظام الرعاية الصحية الأولية الفعا ل | 27 |
|  |  | ضمن منظور الرعاية الصحية الأولية، العديد من الوظائف التي يؤديها حاليًا العاملون في الرعاية الصحية العامة هي من مسؤولية المهنيين الصحيين | 28 |
|  |  | لا تعتبر خدمات الرعاية الحادة جزءًا من نظام الرعاية الصحية الأولية | 29 |
|  |  | في نظام الرعاية الصحية الأولية، تبذل الجهود لاستخدام التكنولوجيا والأفراد الأقل تكلفة لتحقيق نتائج صحية إيجابية | 30 |
|  |  | تركز الرعاية الصحية الأولية على تحديد الأهداف وخطط العمل لتحقيق الأهداف الصحية الوطنية | 31 |
|  |  | أحد المبادئ الخمسة الأساسية للرعاية الصحية الأولية هو توفير رعاية طبية عالية الجودة عرضية | 32 |
|  |  | لا تشمل خدمات الرعاية الصحية الأولية الرعاية العلاجية أو التلطيفية | 33 |
|  |  | نظام الرعاية الصحية الإقليمي الحالي لدينا يستند إلى نموذج الرعاية الصحية الأولية | 34 |
|  |  | تحالف من كبار السن والشرطة والتجار المحليين للعمل على تحسين أمان الحي هو استراتيجية مناسبة للرعاية الصحية الأولية | 35 |

مقياس المواقف
التعليمات:
يتضمن هذا القسم 34 عبارة تتعلق بالمواقف تجاه الرعاية الصحية الأولية. يرجى تحديد مستوى موافقتك مع كل عبارة باستخدام المقياس التالي

غير موافق بشدة

غير موافق

موافق

موافق بشدة

قم بالإشارة إلى الرقم الذي يعبر عن رأيك بصدق لكل عبارة. لا توجد إجابات صحيحة أو خاطئة

| موافق بشدة | موافق | غير موافق | غير موافق بشدة | مقياس المواقف | **الرقم** |
| --- | --- | --- | --- | --- | --- |
|  |  |  |  | يجب تخصيص المزيد من الأموال للرعاية الصحية لتطوير المعدات التكنولوجية لتشخيص الأمراض | **1** |
|  |  |  |  | زيادة التخصص الطبي ضرورية لتحسين صحة المجتمع | **2** |
|  |  |  |  | يجب أن يحصل جميع الناس في أي بلد على رعاية صحية أساسية حتى لو كان ذلك يعني أن بعض الناس سيحصلون على خدمات أقل مما يحصلون عليه حاليً | **3** |
|  |  |  |  | يمكن للممرضين تقديم العديد من خدمات الرعاية الصحية التي يقدمها الأطباء حاليً | **4** |
|  |  |  |  | احتاج معظم الأطفال إلى رعاية صحية وقائية من طبيب الأطفال بدلاً من طبيب عام | **5** |
|  |  |  |  | يجب إلغاء نظام الدفع عن طريق الخدمة للأطباء واستبداله بنظام دفع آخر | **6** |
|  |  |  |  | الحصول على رعاية صحية جيدة هو حق أساسي لجميع الناس | **7** |
|  |  |  |  | يجب إنفاق المزيد من الأموال على تعزيز الصحة والوقاية من الأمراض حتى لو كان ذلك يعني تقليل الأموال المتاحة للعلاج المتخصص والرعاية الحادة | **8** |
|  |  |  |  | يمكن للمتطوعين والعاملين غير المتخصصين تقديم العديد من الخدمات التي يقدمها المهنيون الصحيون حاليًا | **9** |
|  |  |  |  | الطبيب هو أفضل شخص للحفاظ على صحة الناس | **10** |
|  |  |  |  | يمكن للممرضين أداء العديد من المهام التي يقوم بها الأطباء بنفس الكفاءة | **11** |
|  |  |  |  | تعليم الناس كيفية الحفاظ على صحتهم هو دور مهم للممرضين | **12** |
|  |  |  |  | يجب أن يقضي الأطباء والممرضين المزيد من الوقت في تقديم المعلومات للناس حول حالتهم الصحية | **13** |
|  |  |  |  | يجب أن يكون للنساء الحوامل دور متزايد في اتخاذ القرارات المتعلقة بالإجراءات المستخدمة أثناء الولادة | **14** |
|  |  |  |  | يجب بذل المزيد من الجهود من قبل المهنيين الصحيين لتشجيع العلاجات التقليدية كجزء من تقديم الرعاية الصحية | **15** |
|  |  |  |  | يجب إنفاق المزيد من الأموال على الوقاية من الحوادث للأطفال والمراهقين حتى لو أدى ذلك إلى زيادة أوقات الانتظار لبعض العمليات الجراحية الاختيارية | **16** |
|  |  |  |  | يجب أن يتحمل نظام الرعاية الصحية مسؤولية أكبر في تطوير برامج لمعالجة مشكلات مثل العنف الأسري | **17** |
|  |  |  |  | الطريقة لحل مشكلة ارتفاع معدلات الوفاة من أمراض القلب هي فحص الأطفال للكشف عن مستويات الدهون المرتفعة | **18** |
|  |  |  |  | كل شخص مسؤول عن الحفاظ على صحته | **19** |
|  |  |  |  | يجب أن تكون القابلات المدربات متاحات للنساء اللاتي يخترن الاستفادة من خدمات هذه المهنيات | **20** |
|  |  |  |  | يجب أن يُعطى نفس القدر من الاهتمام لمساعدة الناس على التكيف مع مشاكلهم الصحية كما يُعطى للتشخيص والعلاج | **21** |
|  |  |  |  | إشراك المستهلكين في اتخاذ القرارات بشأن كيفية إنفاق أموال الرعاية الصحية سيؤدي إلى انخفاض جودة الرعاية الصحية | **22** |
|  |  |  |  | يجب أن يحصل الناس على وصول واسع للمعلومات الصحية من الأطباء والممرضين ليتمكنوا من المشاركة الكاملة في اتخاذ القرارات التي تؤثر على صحتهم | **23** |
|  |  |  |  | تحتاج الحكومات الإقليمية إلى تحمل المزيد من المسؤولية لضمان حصول الناس في جميع المناطق على الخدمات الطبية | **24** |
|  |  |  |  | الأفراد قادرون على تحمل المسؤولية الأساسية عن صحتهم اللازمة، حتى لو كان ذلك يعني التحكم في مكان عمل بعض الأطبا | **25** |
|  |  |  |  | إذا كان بإمكان الأفراد تحمل تكاليف الرسوم على الخدمات، فيجب أن يكونوا متوقعين القيام بذلك | **26** |
|  |  |  |  | إذا كان بإمكان الأفراد تحمل تكاليف الرسوم على الخدمات، فيجب أن يكونوا متوقعين القيام بذلك | **27** |
|  |  |  |  | الرعاية الصحية الأولية هي نهج مفيد للدول النامية ولكن له أهمية قليلة للدول الصناعية R | **28** |
|  |  |  |  | المؤشرات الصحية المستخدمة حاليًا (مثل المراضة والوفيات) طرق مفيدة ولكنها محدودة لتحديد فعالية الرعاية الصحية الأولية | **29** |
|  |  |  |  | الرعاية الصحية الأولية مناسبة فقط للمجموعات والمجتمعات ذات الدخل المنخفض R | **30** |
|  |  |  |  | يتم المبالغة في التركيز على التباينات الثقافية في ممارسات الرعاية الصحية | **31** |
|  |  |  |  | تعد مشاركة المجتمع عائقًا في تنفيذ برامج الرعاية الصحية الأولية R | **32** |
|  |  |  |  | بغض النظر عن مكان إنفاق أموال الرعاية الصحية، لن نتمكن أبدًا من تقليل معدل الإصابة بالأمراض R | **33** |
|  |  |  |  | جميع المهنيين الصحيين لديهم دور في الرعاية الصحية الأولية | **34** |

نظام التصحيح
غير موافق بشدة تمنح نقطة واحدة
غير موافق تمنح نقطتين
موافق تمنح ثلاث نقاط
موافق بشدة تمنح أربع نقاط
سيتم تصحيح العبارات ذات الصياغة السلبية بشكل عكسي لضمان قياس المواقف بشكل دقيق
إجمالي نطاق الدرجات من 34 إلى 136
