# Supplementary material for: Undergraduate nursing students’ knowledge and attitudes towards Primary Health Care
Source: PLOS Glob Public Health. 2026 Sep 1;6(9):e0007178. doi: 10.1371/journal.pgph.0007178 (PMC13533354; doi:10.1371/journal.pgph.0007178)
Supplement: Supplementary file 6 [file pgph.0007178.s003.doc]

Responses to reviewers

| **Reviewer comment** | **Response** | **modification** |
| --- | --- | --- |
| Does this manuscript meet PLOS Global Public Health’s publication criteria? Is the manuscript technically sound, and do the data support the conclusions? The manuscript must describe methodologically and ethically rigorous research with conclusions that are appropriately drawn based on the data presented.  Reviewer #1: Partly  Reviewer #2: Partly | Thank you for your comment  Revision was done to the manuscript considering all authors' comments, which accordingly improved the manuscript | See all modifications highlighted in yellow |
| Supplementary material  Figures | Figures were changed according to the journal guidelines, and a list of legends was added | Please see the supporting material and page 38 after the references |
| Reviewer 1 | | |
| The authors need to justify using the Primary Health Care Questionnaire (PHCQ). This tool is from 1997. Is it still considered a state-of-the-art tool, given that it is nearly 30 years old? During that time, PHC has changed considerably due to, among other developments, new technologies (including telehealth), shifts from communicable to noncommunicable diseases, the introduction of new cadres of health care workers, expanded understanding of social determinants, etc. | Thank you for your valuable comment. The Primary Health Care Questionnaire (PHCQ), originally developed in 1997, was created based on a thorough review of literature and expert panel input, demonstrating acceptable reliability and validity for assessing knowledge, attitudes, and practices related to PHC. While the instrument is somewhat outdated, this does not automatically invalidate its use, as many fundamental aspects of primary health care, such as accessibility, prevention, community engagement, and equity, remain central to PHC frameworks.  However, it is important to acknowledge that primary health care has evolved substantially over the past three decades  The study aims to align with the original constructs of PHCQ (e.g., basic PHC knowledge and attitudes rather than system performance or digital health integration).  The tool has been previously used or validated in similar populations or settings, supporting comparability. | Sentence was added to Limitations clearly acknowledge that PHCQ may not fully capture contemporary dimensions of PHC, particularly those related to technological advancements and evolving care models.  Line 652 highlighted |
| Also, how does the PHCQ correlate with the Jordanian nursing training curriculum’s content on PHC? Are all of these topics explicitly covered? (In general, the paper needs a clearer and more precise definition of PHC in Jordan.) | Thank you for your valuable comment. While the PHCQ captures core PHC principles that are included in the Jordanian nursing curriculum (e.g., prevention, health education, and basic care delivery), it does not fully reflect newer components of PHC in Jordan, such as digital health, chronic disease management, and interprofessional care. Therefore, it was considered a tool that assesses foundation rather than comprehensive contemporary PHC competencies. | Two paragraphs were added:  One of them is on the introduction page 7, line 166  Second paragraph in the instrument section, line 268, clarifying implicitly why we chose this tool, and we did not forget to mention this in the limitations |
| Also, do all of the PHCQ items have definitive and agreed-upon answers or are some of more opinions, without “correct” answers? (I also wonder about a questionnaire that has all True answers – it seems that this would confuse respondents. The authors should include more justification of the selection of this tool for the study.) | We thank the reviewer for this important comment. The PHCQ includes both knowledge-based items (with correct answers) and attitudinal items (without definitive answers), and therefore functions as a combined measure rather than a purely objective test.  The concern regarding items with predominantly “True” responses is valid, as such a structure may introduce acquiescence bias and reduce the discriminatory power of the instrument. However, the PHCQ was originally designed to reflect core PHC principles, many of which are inherently affirmative statements. for example, emphasizing prevention, equity, and accessibility.  However, it is acknowledged that the questionnaire design, particularly the predominance of positively framed items, may influence response patterns and should be considered when interpreting the findings. | Please see the limitations section  And the recommendation section  Highlighted in yellow |
| The authors do not present convincing evidence that the demographic and educational characteristics cause the different attitudes and levels of knowledge. The language should be adjusted to say these are associations. Furthermore, in some places where the authors claim causality, it based on uncited “research” or problematic or unfounded claims. | Thank you for your comments. Modifications were done adding supporting evidence and clarifying the interpretation | See lines 556-587 |
| I am not a statistician, so cannot comment on the quality of the statistical analysis. However, it was not clear to me whether the intensive statistical analyses were truly necessary to answer the study’s questions. If yes, then the authors need to discuss more about what the correlations suggest about how to target changes/improvements to nursing education to reach the specific demographic segments of students. | Thank you for this insightful comment. The statistical analyses conducted in this study were intended to move beyond simple description and to identify patterns of association between students’ knowledge, attitudes, and their demographic and academic characteristics. This approach allowed us to better understand whether certain student groups may differ in their exposure to or understanding of primary health care (PHC).  I agree that the value of such analyses lies in their practical implications for nursing education. Accordingly, the Discussion and recommendation sections have been strengthened to better interpret these associations in an applied context. The observed correlations suggest that variations in knowledge and attitudes may be linked to differences in educational exposure, academic progression, or prior experience. | Please see the discussion section highlighted and recommendations |
| The recommendations seem very broad. While it is possible for a reader to guess how they might have been extrapolated from the study, they do not all stem directly from the study findings. (For example, the study does not address teaching methods or quality , so why are there recommendations on these topics?) | Thank you for this important observation. I agree that the initial recommendations were too broad and not all were directly derived from the study findings.  In response, we have carefully revised the Recommendations section to ensure that all points are clearly aligned with the study results, particularly the observed variations in students’ knowledge and attitudes toward primary health care (PHC) and their associations with demographic and academic characteristics. | Please see the modified recommendations highlighted  Line 682 |
| Lastly, on a basic level, the paper needs a serious and skilled edit. In particular, the Introduction and Discussion should be revised for clarity about the study’s arguments and logical flow. It also needs to be carefully copyedited to ensure conciseness, standardize verb tenses, catch phrases that are not complete sentences, simplify run-on sentences, and eliminate repetition of sentences. | Thank you  Full revision and editing were done |  |
| - In lines 173-175, the authors describe a professional diploma in PHC—but is this available to nurses or just physicians? If the latter, then this is perhaps an indication of Jordan’s increasing emphasis on PHC but does not directly affect nurses. | Thank you for your comment.  The professional diploma in primary health care (PHC) mentioned in the manuscript is currently primarily targeted toward physicians, particularly general practitioners and family physicians. We agree that, in its current form, this program does not directly involve nursing students or practicing nurses. However, we included this example to illustrate the broader national shift toward strengthening PHC in Jordan, in which universities play a key role. This shift is relevant to nursing education, as it reflects increasing institutional and policy-level emphasis on PHC that may indirectly influence curriculum development and training priorities across health professions, including nursing. | To address the your concern, I have revised the manuscript to clarify that this initiative is physician-focused and to avoid implying direct applicability to nursing education.  Please see the highlighted sentence line 183 |
| How did the respondents in the study compare with the wider population of nursing students? How do we know that it is a representative sample? | Thank you for your valuable comment. We acknowledge that the use of a convenience sampling method limits the ability to claim full representativeness of the wider population of nursing students in Jordan. However, efforts were made to enhance the diversity of the sample by recruiting participants from multiple universities (both public and private) in central Jordan and by including students at advanced stages of their training (third year and above), who are more likely to have been exposed to primary health care concepts. These steps were intended to provide a broader cross-section of undergraduate nursing students with relevant educational exposure.  In terms of characteristics, the study sample included variation in key demographic and academic variables (e.g., age, gender, academic level), which are generally reflective of the nursing student population. Nevertheless, we did not perform a formal comparison with national-level data on nursing students and therefore cannot definitively conclude that the sample is fully representative.  We clarified this point in the manuscript and emphasized that the findings should be interpreted with caution, particularly in terms of generalizability. Future studies using probability sampling methods and broader geographic coverage across Jordan would strengthen representativeness. | Please see the limitation section, line 675, and the recommendation section, line 704 |
| What is meant by “intent to leave nursing in the future”? Are these people understood to be leaving health care all together or as seeking additional training in a health care specialization? | Thank you for this important point. The “intent to leave nursing in the future” refers specifically to students’ self-reported plans regarding their intention to exit the nursing profession entirely after graduation, rather than pursuing further education or specialization within health care. Students who indicated an intent to leave were considered as expressing a potential future disengagement from the nursing workforce.  This distinction is important, as it may be associated with differences in knowledge and attitudes toward primary health care, reflecting either reduced engagement or motivation within the nursing field. | A sentence was added to the instrument section defining this variable line 260 |
| I did not understand Table 3—how was this sub-sample of n=108 selected? | The sub-sample of n = 108 in Table 3 represents the participants who correctly completed all items related to the knowledge section of the PHC questionnaire. While the total study sample was 157 participants, 49 students had wrong answers and were therefore excluded from the calculation of the knowledge score to ensure accuracy. | In the above table, the title clarifies that these results are only for correct answers. All statistical analyses were done according to the original tool authors' recommendations in their paper. |
| In lines 433-435, the study states that “males had a higher mean score of positive attitudes towards primary health care”, but in line 586, the authors state “female students are more likely to have positive attitudes…”. This seems contradictory. Also, the latter statement says “Some research indicates…” but does not actually provide any citations. | Thank you for your comment. You are right indeed. Modifications were done | Please see the discussion section, lines 570-577 |
| The paper sometimes lacks consistent and clear terminology. It mentions (in the Abstract and Implications sections) “practical research,” but it is not clear whether it specifically means this study. Another example is in line 113 which mentions nursing students’ opinions about “public health” – this is not the same as primary health care. Finally, lines 529 and 609 comment on students’ attitudes about “medical procedures” – this is the first time this is mentioned, and it is not clear where this comes from. | Thank you for this comment. The sentences and terminology were reviewed, and clear rephrasing was done  Yes, you are right in the finding, and according to specific items, they had positive attitude toward the medical services provided in the PHC | See abstract line 42  Implications line 649  Modifications were done and highlighted |
| Reviewer 2 | | |
| Introduction: 1. The manuscript claims to be the first of its kind in Jordan assessing nursing students’ knowledge and attitudes toward PHC. However, the introduction cites Abu-Baker et al. (2022) and Al Ali et al. (2017), which already explored related aspects of health promotion and awareness in Jordan. Clarify the novelty by explicitly distinguishing this study from prior Jordanian research (e.g., focus on undergraduate nursing students, use of PHCQ, or predictive modeling of attitudes). Strengthen the justification for why this study fills a gap. | Thank you for your comment.  You are right. A short paragraph was added in the introduction focus on the novelty of this study compared to Ali and Abubaker | Please see line 197 |
| 2. The introdcution is unneccesary long. Could the authors make it very succinct. | The review was done |  |
| Methodology: 1. Sampling: The study uses convenience sampling but also mentions “clustered stratified random sampling” when selecting universities. This is inconsistent. Clearly state the sampling strategy. If convenience sampling was used for students, explain why, and discuss limitations (e.g., potential bias, limited generalizability). | Thank you for your comment.  To clarify, a multi-stage sampling approach was used in this study:  First, universities were selected using a clustered stratified random sampling method, where institutions were stratified by type (governmental vs. private), and four universities (two from each stratum) in central Jordan were randomly selected.  Second, within the selected universities, students were recruited using convenience sampling, based on eligibility criteria (undergraduate nursing students in their third year or above) and voluntary participation.  We have revised the manuscript to clearly distinguish between these two stages of sampling.  We also acknowledge that the use of convenience sampling at the student level may introduce selection bias and limit generalizability, as participants who chose to respond may differ from those who did not. This limitation has now been explicitly stated in the revised manuscript. | Please see line 243 in the setting section  Line 691 in limitation |
| 2. Statistical Analysis: Regression results are reported with coefficients (B=0.173, B=0.044, B=0.122) but without confidence intervals or full regression tables. Kindly provide detailed statistical outputs (e.g., beta coefficients, confidence intervals, p-values, R²). This transparency is required by PLOS standards. | Confidence interval was added in table 7 and R^2^ in table 8 | Please see highlighted numbers in table 7 and 8 |
| 3. Ethical Considerations: Ethical approval is mentioned briefly, but no institutional review board (IRB) approval number or committee details are provided. Add the IRB approval reference, committee name, and approval date to meet journal requirements. | Thank you for your comment.  The IRB committee name and reference number mentioned in line 327 and 725 | Please see mentioned lines |
| Data Availability: The statement says data are available “upon request.” PLOS requires open data availability unless legal/ethical restrictions apply. Deposit the dataset and Arabic PHCQ in a public repository (e.g., Figshare, Dryad) and provide a DOI. If restrictions exist, justify them explicitly. | Thank you for your comment. And according to journal policy the statement was changed please see data availability | Modified statement  The datasets generated and/or analyzed during the current study, as well as the Arabic version of the questionnaire, are publicly available.  An Arabic tool was attached to the supported materials |
| Discussion: The discussion reiterates results but does not critically compare findings with international literature (e.g., Bloomfield et al. 2015, Khattak et al. 2022). Expand the discussion to highlight similarities/differences with global studies, and explain implications for nursing curricula in Jordan and beyond. | Thank you for your comments  We agree that the original Discussion section relied heavily on reiterating the study findings and did not sufficiently engage with international literature.  In response, we have revised the Discussion to:  Incorporate comparisons with international studies (e.g., Bloomfield et al., 2015; Khattak et al., 2022)  Highlight similarities and differences across contexts  Provide clearer interpretation of findings in relation to nursing education in Jordan and globally and all modifications were highlighted | Please see highlighted paragraphs |
| Some sentences are long and repetitive (e.g., “PHC’s main responsibilities according to WHO include…”). Suggestion: Shorten sentences, make them succinct and avoid redundancy for readability. | Thank you  The sensence was summarized | Please see line 58 |
| Use consistent terms: sometimes “PHC nurses,” sometimes “nursing students in PHC.” Standardize terminology throughout. | All terminology was reviewed, and consistency was maintained according to the context | All modifications in yellow |
| No tables or figures are included in the excerpt. PLOS requires clear presentation of descriptive statistics and regression results. Add tables summarizing demographics, knowledge/attitude levels, and regression outputs. | Thank you for your comment.  8 tables are presented in the main manuscript and 2 figures with a detailed description  And according to the journal guidelines, a separate document for figures was added | Please see the tables and figures  And there are legends at the end of the manuscript that describe exactly what is presented in the figures |
| Some references (e.g., Allari et al., 2025) appear to be unpublished or future-dated. Please verify and update references to ensure accuracy and credibility. | This reference was verified  It was published and scopus indexed | <https://journals.sagepub.com/doi/full/10.1177/23779608251340682> |
| Minor grammatical issues (e.g., “the study through SPSS reverses the codes of those items” ---to--- “the study reversed item codes using SPSS”). Kindly proofread for grammar and style consistency. | Thank you for your valuable notes. Yeas you are right I modified the grammatical errors  Proof reading was done | Please see line 290, 422 |
