# Supplementary material for: Undergraduate nursing students’ knowledge and attitudes towards Primary Health Care
Source: PLOS Glob Public Health. 2026 Sep 1;6(9):e0007178. doi: 10.1371/journal.pgph.0007178 (PMC13533354; doi:10.1371/journal.pgph.0007178)
Supplement: Peer review history

# Peer review: Decision Letter 0

**DOI:** 10.1371/journal.pgph.0007178.r001

**Authors:** Pengpeng Ye

12 Mar 2026

PGPH-D-25-02221

Undergraduate Nursing Students’ Knowledge and Attitudes Towards Primary Health Care

PLOS Global Public Health

Dear Dr. Allari,

Thank you for submitting your manuscript to PLOS Global Public Health. After careful consideration, we feel that it has merit but does not fully meet PLOS Global Public Health’s publication criteria as it currently stands. Therefore, we invite you to submit a revised version of the manuscript that addresses the points raised during the review process.

Please submit your revised manuscript by Apr 26 2026 11:59PM. If you will need more time than this to complete your revisions, please reply to this message or contact the journal office at globalpubhealth@plos.org. When you're ready to submit your revision, log on to https://www.editorialmanager.com/pgph/ and select the 'Submissions Needing Revision' folder to locate your manuscript file.

Please include the following items when submitting your revised manuscript:

- A letter that responds to each point raised by the editor and reviewer(s). You should upload this letter as a separate file labeled 'Response to Reviewers'.
- A marked-up copy of your manuscript that highlights changes made to the original version. You should upload this as a separate file labeled 'Revised Manuscript with Track Changes'.
- An unmarked version of your revised paper without tracked changes. You should upload this as a separate file labeled 'Manuscript'.

Guidelines for resubmitting your figure files are available below the reviewer comments at the end of this letter.

We look forward to receiving your revised manuscript.

Kind regards,

Pengpeng Ye

Academic Editor

PLOS Global Public Health

Journal Requirements:

1. Please provide separate figure files in .tif or .eps format.

For more information about figure files please see our guidelines:  LINK

https://journals.plos.org/globalpublichealth/s/figures

https://journals.plos.org/globalpublichealth/s/figures#loc-file-requirements

2. We have noticed that you have uploaded Supporting Information files, but you have not included a list of legends. Please add a full list of legends for your Supporting Information files after the references list.

If the reviewer comments include a recommendation to cite specific previously published works, please review and evaluate these publications to determine whether they are relevant and should be cited. There is no requirement to cite these works unless the editor has indicated otherwise.

Additional Editor Comments (if provided):

[Note: HTML markup is below. Please do not edit.]

Reviewers' comments:

Reviewer's Responses to Questions

**Comments to the Author**

1. Does this manuscript meet PLOS Global Public Health’s publication criteria?

Reviewer #1: Partly

Reviewer #2: Partly

**********

2. Has the statistical analysis been performed appropriately and rigorously?

Reviewer #1: I don't know

Reviewer #2: Yes

**********

3. Have the authors made all data underlying the findings in their manuscript fully available (please refer to the Data Availability Statement at the start of the manuscript PDF file)?

The PLOS Data policy

Reviewer #1: Yes

Reviewer #2: Yes

**********

4. Is the manuscript presented in an intelligible fashion and written in standard English?

Reviewer #1: No

Reviewer #2: No

**********

Reviewer #1: The authors of this study surveyed Jordanian nursing students on their knowledge of and attitudes about primary health care (PHC). They conducted statistical analyses on the survey data to look for correlations between academic and demographic factors with PHC knowledge and attitudes. The overarching purpose for conducting the study seems to be to support efforts to continue increasing the focus on PHC in Jordan by examining whether and how well nursing training is contributing to the PHC workforce. This study touches on numerous important topics and begins to address gaps in the literature on nursing education in Jordan.

However, the paper is not ready for publication at this time.

Here are some big picture questions/concerns:

- The authors need to justify using the Primary Health Care Questionnaire (PHCQ). This tool is from 1997. Is it still considered a state-of-the-art tool, given that it is nearly 30 years old? During that time, PHC has changed considerably due to, among other developments, new technologies (including telehealth), shifts from communicable to noncommunicable diseases, the introduction of new cadres of health care workers, expanded understanding of social determinants, etc.

Also, how does the PHCQ correlate with the Jordanian nursing training curriculum’s content on PHC? Are all of these topics explicitly covered? (In general, the paper needs a clearer and more precise definition of PHC in Jordan.)

Also, do all of the PHCQ items have definitive and agreed-upon answers or are some of more opinions, without “correct” answers? (I also wonder about a questionnaire that has all True answers – it seems that this would confuse respondents. The authors should include more justification of the selection of this tool for the study.)

- The authors do not present convincing evidence that the demographic and educational characteristics cause the different attitudes and levels of knowledge. The language should be adjusted to say these are associations. Furthermore, in some places where the authors claim causality, it based on uncited “research” or problematic or unfounded claims.

- I am not a statistician, so cannot comment on the quality of the statistical analysis. However, it was not clear to me whether the intensive statistical analyses were truly necessary to answer the study’s questions. If yes, then the authors need to discuss more about what the correlations suggest about how to target changes/improvements to nursing education to reach the specific demographic segments of students.

- The recommendations seem very broad. While it is possible for a reader to guess how they might have been extrapolated from the study, they do not all stem directly from the study findings. (For example, the study does not address teaching methods or quality , so why are there recommendations on these topics?)

- Lastly, on a basic level, the paper needs a serious and skilled edit. In particular, the Introduction and Discussion should be revised for clarity about the study’s arguments and logical flow. It also needs to be carefully copyedited to ensure conciseness, standardize verb tenses, catch phrases that are not complete sentences, simplify run-on sentences, and eliminate repetition of sentences.

And here are some specific questions:

- In lines 173-175, the authors describe a professional diploma in PHC—but is this available to nurses or just physicians? If the latter, then this is perhaps an indication of Jordan’s increasing emphasis on PHC but does not directly affect nurses.

- How did the respondents in the study compare with the wider population of nursing students? How do we know that it is a representative sample?

- What is meant by “intent to leave nursing in the future”? Are these people understood to be leaving health care all together or as seeking additional training in a health care specialization?

- I did not understand Table 3—how was this sub-sample of n=108 selected?

- In lines 433-435, the study states that “males had a higher mean score of positive attitudes towards primary health care”, but in line 586 the authors state “female students are more likely to have positive attitudes…”. This seems contradictory. Also, the latter statement says “Some research indicates…” but does not actually provide any citations.

- The paper sometimes lacks consistent and clear terminology. It mentions (in the Abstract and Implications sections) “practical research,” but it is not clear whether it specifically means this study. Another example is in line 113 which mentions nursing students’ opinions about “public health” – this is not the same as primary health care. Finally, lines 529 and 609 comment on students’ attitudes about “medical procedures” – this is the first time this is mentioned, and it is not clear where this comes from.

Reviewer #2: Reviewer comments

Study title: Undergraduate Nursing Students’ Knowledge and Attitudes Towards Primary Health Care

General comments:

This is an interesting study. It reflects the knowledge level of students relating to primary health care.

Major Comments

Introduction:

1. The manuscript claims to be the first of its kind in Jordan assessing nursing students’ knowledge and attitudes toward PHC. However, the introduction cites Abu-Baker et al. (2022) and Al Ali et al. (2017), which already explored related aspects of health promotion and awareness in Jordan. Clarify the novelty by explicitly distinguishing this study from prior Jordanian research (e.g., focus on undergraduate nursing students, use of PHCQ, or predictive modeling of attitudes). Strengthen the justification for why this study fills a gap.

2. The introdcution is unneccesary long. Could the authors make it very succinct.

Methodology:

1. Sampling: The study uses convenience sampling but also mentions “clustered stratified random sampling” when selecting universities. This is inconsistent. Clearly state the sampling strategy. If convenience sampling was used for students, explain why, and discuss limitations (e.g., potential bias, limited generalizability).

2. Statistical Analysis: Regression results are reported with coefficients (B=0.173, B=0.044, B=0.122) but without confidence intervals or full regression tables. Kindly provide detailed statistical outputs (e.g., beta coefficients, confidence intervals, p-values, R²). This transparency is required by PLOS standards.

3. Ethical Considerations: Ethical approval is mentioned briefly, but no institutional review board (IRB) approval number or committee details are provided. Add the IRB approval reference, committee name, and approval date to meet journal requirements.

Data Availability:

The statement says data are available “upon request.” PLOS requires open data availability unless legal/ethical restrictions apply. Deposit the dataset and Arabic PHCQ in a public repository (e.g., Figshare, Dryad) and provide a DOI. If restrictions exist, justify them explicitly.

Discussion:

The discussion reiterates results but does not critically compare findings with international literature (e.g., Bloomfield et al. 2015, Khattak et al. 2022). Expand the discussion to highlight similarities/differences with global studies, and explain implications for nursing curricula in Jordan and beyond.

Minor Comments

1. Some sentences are long and repetitive (e.g., “PHC’s main responsibilities according to WHO include…”). Suggestion: Shorten sentences, make them succinct and avoid redundancy for readability.

2. Use consistent terms: sometimes “PHC nurses,” sometimes “nursing students in PHC.” Standardize terminology throughout.

3. No tables or figures are included in the excerpt. PLOS requires clear presentation of descriptive statistics and regression results. Add tables summarizing demographics, knowledge/attitude levels, and regression outputs.

4. Some references (e.g., Allari et al., 2025) appear to be unpublished or future-dated. Please verify and update references to ensure accuracy and credibility.

5. Minor grammatical issues (e.g., “the study through SPSS reverses the codes of those items” ---to--- “the study reversed item codes using SPSS”). Kindly proofread for grammar and style consistency.

**********

what does this mean?). If published, this will include your full peer review and any attached files.

**Do you want your identity to be public for this peer review?**  If you choose “no”, your identity will remain anonymous but your review may still be made public.

For information about this choice, including consent withdrawal, please see our Privacy Policy

Reviewer #1: No

Reviewer #2: No

**********

[NOTE: If reviewer comments were submitted as an attachment file, they will be attached to this email and accessible via the submission site. Please log into your account, locate the manuscript record, and check for the action link "View Attachments". If this link does not appear, there are no attachment files.]

# Author comment: Author response to Decision Letter 1

**DOI:** 10.1371/journal.pgph.0007178.r002

8 Apr 2026

Attachment Submitted filename: response.doc

# Peer review: Decision Letter 1

**DOI:** 10.1371/journal.pgph.0007178.r003

**Authors:** Pengpeng Ye

2 Jun 2026

PGPH-D-25-02221R1

Undergraduate Nursing Students’ Knowledge and Attitudes Towards Primary Health Care

PLOS Global Public Health

Dear Dr. Allari,

Thank you for submitting your manuscript to PLOS Global Public Health. After careful consideration, we feel that it has merit but does not fully meet PLOS Global Public Health’s publication criteria as it currently stands. Therefore, we invite you to submit a revised version of the manuscript that addresses the points raised during the review process.

Please submit your revised manuscript by Jul 02 2026 11:59PM. If you will need more time than this to complete your revisions, please reply to this message or contact the journal office at globalpubhealth@plos.org. When you're ready to submit your revision, log on to https://www.editorialmanager.com/pgph/ and select the 'Submissions Needing Revision' folder to locate your manuscript file.

Please include the following items when submitting your revised manuscript:

- A letter that responds to each point raised by the editor and reviewer(s). You should upload this letter as a separate file labeled 'Response to Reviewers'.
- A marked-up copy of your manuscript that highlights changes made to the original version. You should upload this as a separate file labeled 'Revised Manuscript with Track Changes'.
- An unmarked version of your revised paper without tracked changes. You should upload this as a separate file labeled 'Manuscript'.

Guidelines for resubmitting your figure files are available below the reviewer comments at the end of this letter.

As the corresponding author, your ORCID iD is verified in the submission system and will appear in the published article. PLOS supports the use of ORCID, and we encourage all coauthors to register for an ORCID iD and use it as well. Please encourage your coauthors to verify their ORCID iD within the submission system before final acceptance, as unverified ORCID iDs will not appear in the published article. *Only*  the individual author can complete the verification step; PLOS staff *cannot*  verify ORCID iDs on behalf of authors.

We look forward to receiving your revised manuscript.

Kind regards,

Pengpeng Ye

Academic Editor

PLOS Global Public Health

Journal Requirements:

If the reviewer comments include a recommendation to cite specific previously published works, please review and evaluate these publications to determine whether they are relevant and should be cited. There is no requirement to cite these works unless the editor has indicated otherwise.

Please review your reference list to ensure that it is complete and correct. If you have cited papers that have been retracted, please include the rationale for doing so in the manuscript text, or remove these references and replace them with relevant current references. Any changes to the reference list should be mentioned in the rebuttal letter that accompanies your revised manuscript. If you need to cite a retracted article, indicate the article’s retracted status in the References list and also include a citation and full reference for the retraction notice.

Additional Editor Comments (if provided):

[Note: HTML markup is below. Please do not edit.]

Reviewers' comments:

Reviewer's Responses to Questions

**Comments to the Author**

Reviewer #1: All comments have been addressed

**********

publication criteria?

Reviewer #1: Yes

**********

3. Has the statistical analysis been performed appropriately and rigorously?

Reviewer #1: Yes

**********

4. Have the authors made all data underlying the findings in their manuscript fully available (please refer to the Data Availability Statement at the start of the manuscript PDF file)?

The PLOS Data policy

Reviewer #1: Yes

**********

5. Is the manuscript presented in an intelligible fashion and written in standard English?

Reviewer #1: No

**********

Reviewer #1: The paper still requires a careful and comprehensive language edit.

**********

what does this mean?). If published, this will include your full peer review and any attached files.

**Do you want your identity to be public for this peer review?**  If you choose “no”, your identity will remain anonymous but your review may still be made public.

For information about this choice, including consent withdrawal, please see our Privacy Policy

Reviewer #1: No

**********

[NOTE: If reviewer comments were submitted as an attachment file, they will be attached to this email and accessible via the submission site. Please log into your account, locate the manuscript record, and check for the action link "View Attachments". If this link does not appear, there are no attachment files.]

# Author comment: Author response to Decision Letter 2

**DOI:** 10.1371/journal.pgph.0007178.r004

4 Jun 2026

Attachment Submitted filename: responses.docx

# Peer review: Decision Letter 2

**DOI:** 10.1371/journal.pgph.0007178.r005

**Authors:** Pengpeng Ye

18 Jun 2026

PGPH-D-25-02221R2

Undergraduate Nursing Students’ Knowledge and Attitudes Towards Primary Health Care

PLOS Global Public Health

Dear Dr. Allari,

Thank you for submitting your manuscript to PLOS Global Public Health. After careful consideration, we feel that it has merit but does not fully meet PLOS Global Public Health’s publication criteria as it currently stands. Therefore, we invite you to submit a revised version of the manuscript that addresses the points raised during the review process.

Please submit your revised manuscript by Jul 18 2026 11:59PM. If you will need more time than this to complete your revisions, please reply to this message or contact the journal office at globalpubhealth@plos.org. When you're ready to submit your revision, log on to https://www.editorialmanager.com/pgph/ and select the 'Submissions Needing Revision' folder to locate your manuscript file.

Please include the following items when submitting your revised manuscript:

- A letter that responds to each point raised by the editor and reviewer(s). You should upload this letter as a separate file labeled 'Response to Reviewers'.
- A marked-up copy of your manuscript that highlights changes made to the original version. You should upload this as a separate file labeled 'Revised Manuscript with Track Changes'.
- An unmarked version of your revised paper without tracked changes. You should upload this as a separate file labeled 'Manuscript'.

Guidelines for resubmitting your figure files are available below the reviewer comments at the end of this letter.

As the corresponding author, your ORCID iD is verified in the submission system and will appear in the published article. PLOS supports the use of ORCID, and we encourage all coauthors to register for an ORCID iD and use it as well. Please encourage your coauthors to verify their ORCID iD within the submission system before final acceptance, as unverified ORCID iDs will not appear in the published article. *Only*  the individual author can complete the verification step; PLOS staff *cannot*  verify ORCID iDs on behalf of authors.

We look forward to receiving your revised manuscript.

Kind regards,

Pengpeng Ye

Academic Editor

PLOS Global Public Health

Journal Requirements:

1. If the reviewer comments include a recommendation to cite specific previously published works, please review and evaluate these publications to determine whether they are relevant and should be cited. There is no requirement to cite these works unless the editor has indicated otherwise.

2. Please review your reference list to ensure that it is complete and correct. If you have cited papers that have been retracted, please include the rationale for doing so in the manuscript text, or remove these references and replace them with relevant current references. Any changes to the reference list should be mentioned in the rebuttal letter that accompanies your revised manuscript. If you need to cite a retracted article, indicate the article’s retracted status in the References list and also include a citation and full reference for the retraction notice.

Additional Editor Comments (if provided):

[Note: HTML markup is below. Please do not edit.]

Reviewers' comments:

Reviewer's Responses to Questions

**Comments to the Author**

Reviewer #1: (No Response)

**********

publication criteria?

Reviewer #1: Partly

**********

3. Has the statistical analysis been performed appropriately and rigorously?

Reviewer #1: N/A

**********

4. Have the authors made all data underlying the findings in their manuscript fully available (please refer to the Data Availability Statement at the start of the manuscript PDF file)?

The PLOS Data policy

Reviewer #1: Yes

**********

5. Is the manuscript presented in an intelligible fashion and written in standard English?

Reviewer #1: No

**********

Reviewer #1: I'm sorry to say that I still do not think this manuscript is ready to be accepted and published. The minimal changes made do not address many of the inconsistencies in the manuscript's language. There are problems with inconsistent tenses, misused transition words, and word choice. These challenges make many of the paper's findings difficult to understand and assess for validity.

It could be published the way it is in a less reputable journal -- I definitely have read many papers with equally awkward language. But you risk having your messages misunderstood or undervalued because they are not being communicated in a clear and professional manner.

**********

what does this mean?). If published, this will include your full peer review and any attached files.

**Do you want your identity to be public for this peer review?**  If you choose “no”, your identity will remain anonymous but your review may still be made public.

For information about this choice, including consent withdrawal, please see our Privacy Policy

Reviewer #1: No

**********

[NOTE: If reviewer comments were submitted as an attachment file, they will be attached to this email and accessible via the submission site. Please log into your account, locate the manuscript record, and check for the action link "View Attachments". If this link does not appear, there are no attachment files.]

# Author comment: Author response to Decision Letter 3

**DOI:** 10.1371/journal.pgph.0007178.r006

28 Jun 2026

Attachment Submitted filename: responses 28-6.docx

# Editor report: Decision Letter 3

**DOI:** 10.1371/journal.pgph.0007178.r007

**Authors:** Pengpeng Ye

2 Jul 2026

PGPH-D-25-02221R3

Undergraduate Nursing Students’ Knowledge and Attitudes Towards Primary Health Care

PLOS Global Public Health

Dear Dr. Allari,

Thank you for submitting your manuscript to PLOS Global Public Health. After careful consideration, we feel that it has merit but does not fully meet PLOS Global Public Health’s publication criteria as it currently stands. Therefore, we invite you to submit a revised version of the manuscript that addresses the points raised during the review process.

Please submit your revised manuscript by Aug 01 2026 11:59PM. If you will need more time than this to complete your revisions, please reply to this message or contact the journal office at globalpubhealth@plos.org. When you're ready to submit your revision, log on to https://www.editorialmanager.com/pgph/ and select the 'Submissions Needing Revision' folder to locate your manuscript file.

Please include the following items when submitting your revised manuscript:

- A letter that responds to each point raised by the editor and reviewer(s). You should upload this letter as a separate file labeled 'Response to Reviewers'.
- A marked-up copy of your manuscript that highlights changes made to the original version. You should upload this as a separate file labeled 'Revised Manuscript with Track Changes'.
- An unmarked version of your revised paper without tracked changes. You should upload this as a separate file labeled 'Manuscript'.

Guidelines for resubmitting your figure files are available below the reviewer comments at the end of this letter.

As the corresponding author, your ORCID iD is verified in the submission system and will appear in the published article. PLOS supports the use of ORCID, and we encourage all coauthors to register for an ORCID iD and use it as well. Please encourage your coauthors to verify their ORCID iD within the submission system before final acceptance, as unverified ORCID iDs will not appear in the published article. *Only*  the individual author can complete the verification step; PLOS staff *cannot*  verify ORCID iDs on behalf of authors.

We look forward to receiving your revised manuscript.

Kind regards,

Pengpeng Ye

Academic Editor

PLOS Global Public Health

Journal Requirements:

If the reviewer comments include a recommendation to cite specific previously published works, please review and evaluate these publications to determine whether they are relevant and should be cited. There is no requirement to cite these works unless the editor has indicated otherwise.

Please review your reference list to ensure that it is complete and correct. If you have cited papers that have been retracted, please include the rationale for doing so in the manuscript text, or remove these references and replace them with relevant current references. Any changes to the reference list should be mentioned in the rebuttal letter that accompanies your revised manuscript. If you need to cite a retracted article, indicate the article’s retracted status in the References list and also include a citation and full reference for the retraction notice.

Additional Editor Comments (if provided):

[Note: HTML markup is below. Please do not edit.]

Reviewers' comments:

[NOTE: If reviewer comments were submitted as an attachment file, they will be attached to this email and accessible via the submission site. Please log into your account, locate the manuscript record, and check for the action link "View Attachments". If this link does not appear, there are no attachment files.]

# Author comment: Author response to Decision Letter 4

**DOI:** 10.1371/journal.pgph.0007178.r008

3 Jul 2026

Attachment Submitted filename: responses 3-7-2026.docx

# Peer review: Decision Letter 4

**DOI:** 10.1371/journal.pgph.0007178.r009

**Authors:** Pengpeng Ye

17 Jul 2026

PGPH-D-25-02221R4

Undergraduate Nursing Students’ Knowledge and Attitudes Towards Primary Health Care

PLOS Global Public Health

Dear Dr. Allari,

Thank you for submitting your manuscript to PLOS Global Public Health. After careful consideration, we feel that it has merit but does not fully meet PLOS Global Public Health’s publication criteria as it currently stands. Therefore, we invite you to submit a revised version of the manuscript that addresses the points raised during the review process.

Reviewer 1 has returned a further set of minor comments. Having carefully considered all reviewers' feedback from this latest round, I believe the remaining points are minor and do not affect the overall scientific validity or contribution of the work. On this basis, I recommend that the manuscript be provisionally accepted with minor revisions.

Please submit your revised manuscript by Aug 16 2026 11:59PM. If you will need more time than this to complete your revisions, please reply to this message or contact the journal office at globalpubhealth@plos.org. When you're ready to submit your revision, log on to https://www.editorialmanager.com/pgph/ and select the 'Submissions Needing Revision' folder to locate your manuscript file.

Please include the following items when submitting your revised manuscript:

- A letter that responds to each point raised by the editor and reviewer(s). You should upload this letter as a separate file labeled 'Response to Reviewers'.
- A marked-up copy of your manuscript that highlights changes made to the original version. You should upload this as a separate file labeled 'Revised Manuscript with Track Changes'.
- An unmarked version of your revised paper without tracked changes. You should upload this as a separate file labeled 'Manuscript'.

Guidelines for resubmitting your figure files are available below the reviewer comments at the end of this letter.

As the corresponding author, your ORCID iD is verified in the submission system and will appear in the published article. PLOS supports the use of ORCID, and we encourage all coauthors to register for an ORCID iD and use it as well. Please encourage your coauthors to verify their ORCID iD within the submission system before final acceptance, as unverified ORCID iDs will not appear in the published article. *Only*  the individual author can complete the verification step; PLOS staff *cannot*  verify ORCID iDs on behalf of authors.

We look forward to receiving your revised manuscript.

Kind regards,

Pengpeng Ye

Academic Editor

PLOS Global Public Health

Journal Requirements:

If the reviewer comments include a recommendation to cite specific previously published works, please review and evaluate these publications to determine whether they are relevant and should be cited. There is no requirement to cite these works unless the editor has indicated otherwise.

Please review your reference list to ensure that it is complete and correct. If you have cited papers that have been retracted, please include the rationale for doing so in the manuscript text, or remove these references and replace them with relevant current references. Any changes to the reference list should be mentioned in the rebuttal letter that accompanies your revised manuscript. If you need to cite a retracted article, indicate the article’s retracted status in the References list and also include a citation and full reference for the retraction notice.

Additional Editor Comments (if provided):

[Note: HTML markup is below. Please do not edit.]

Reviewers' comments:

Reviewer's Responses to Questions

**Comments to the Author**

Reviewer #1: (No Response)

**********

publication criteria?

Reviewer #1: Partly

**********

3. Has the statistical analysis been performed appropriately and rigorously?

Reviewer #1: I don't know

**********

4. Have the authors made all data underlying the findings in their manuscript fully available (please refer to the Data Availability Statement at the start of the manuscript PDF file)?

The PLOS Data policy

Reviewer #1: Yes

**********

5. Is the manuscript presented in an intelligible fashion and written in standard English?

Reviewer #1: Yes

**********

Reviewer #1: This writing in this paper is improved. However, its messages are still not clear enough to merit publication. I think it tries to combine too many topics, becoming wordy and lacking clarity on messaging. Just a few examples:

- Lines 84-86: it cites nurses as "advocates of the nursing discipline" but then goes on to talk about nurses as advocating "for community health". These are very different endeavors. Which is being addressed here?

- Lines 109 and 157 cite the same statistic (but only once does it clarify that this study was with Australian nursing students).

- Lines 501/505: are these predictors or associations?

- Line 534 There is a disconnected phrase " Before gathering data from 157 students enrolled in the nursing major."

- Lines 576-580 and 674-5 say that students exhibited positive attitudes concerning medical services in PHC and PHC services. What is the difference?

I also do not think that the conclusions of the Abstract match the paper's findings and conclusions.

I would not recommend publication until the paper has been further streamlined and its arguments clarified and honed.

**********

what does this mean?). If published, this will include your full peer review and any attached files.

**Do you want your identity to be public for this peer review?**  If you choose “no”, your identity will remain anonymous but your review may still be made public.

For information about this choice, including consent withdrawal, please see our Privacy Policy

Reviewer #1: No

**********

[NOTE: If reviewer comments were submitted as an attachment file, they will be attached to this email and accessible via the submission site. Please log into your account, locate the manuscript record, and check for the action link "View Attachments". If this link does not appear, there are no attachment files.]

# Author comment: Author response to Decision Letter 5

**DOI:** 10.1371/journal.pgph.0007178.r010

22 Jul 2026

Attachment Submitted filename: response letter 22-7.docx

# Peer review: Decision Letter 5

**DOI:** 10.1371/journal.pgph.0007178.r011

**Authors:** Pengpeng Ye

9 Aug 2026

Undergraduate Nursing Students’ Knowledge and Attitudes Towards Primary Health Care

PGPH-D-25-02221R5

Dear professor Allari,

We are pleased to inform you that your manuscript 'Undergraduate Nursing Students’ Knowledge and Attitudes Towards Primary Health Care' has been provisionally accepted for publication in PLOS Global Public Health.

Before your manuscript can be formally accepted you will need to complete some formatting changes, which you will receive in a follow up email. A member of our team will be in touch with a set of requests.

Please note that your manuscript will not be scheduled for publication until you have made the required changes, so a swift response is appreciated.

IMPORTANT: The editorial review process is now complete. PLOS will only permit corrections to spelling, formatting or significant scientific errors from this point onwards. Requests for major changes, or any which affect the scientific understanding of your work, will cause delays to the publication date of your manuscript.

If your institution or institutions have a press office, please notify them about your upcoming paper to help maximize its impact. If they'll be preparing press materials, please inform our press team as soon as possible -- no later than 48 hours after receiving the formal acceptance. Your manuscript will remain under strict press embargo until 2 pm Eastern Time on the date of publication. For more information, please contact globalpubhealth@plos.org.

Thank you again for supporting Open Access publishing; we are looking forward to publishing your work in PLOS Global Public Health.

Best regards,

Pengpeng Ye

Academic Editor

PLOS Global Public Health

***********************************************************

Reviewer Comments (if any, and for reference):

Reviewer's Responses to Questions

**Comments to the Author**

Reviewer #1: All comments have been addressed

**********

publication criteria?

Reviewer #1: Yes

**********

3. Has the statistical analysis been performed appropriately and rigorously?

Reviewer #1: I don't know

**********

4. Have the authors made all data underlying the findings in their manuscript fully available (please refer to the Data Availability Statement at the start of the manuscript PDF file)?

The PLOS Data policy

Reviewer #1: Yes

**********

5. Is the manuscript presented in an intelligible fashion and written in standard English?

Reviewer #1: Yes

**********

Reviewer #1: It still has a few typos (starting with HealthCare) in the first line and formatting errors.

**********

what does this mean?). If published, this will include your full peer review and any attached files.

**Do you want your identity to be public for this peer review?**  If you choose “no”, your identity will remain anonymous but your review may still be made public.

For information about this choice, including consent withdrawal, please see our Privacy Policy

Reviewer #1: No

**********
